# Two pathogenesis-related proteins interact with leucine-rich repeat proteins to promote *Alternaria* leaf spot resistance in apple

**DOI:** 10.1038/s41438-021-00654-4

**Published:** 2021-10-01

**Authors:** Qiulei Zhang, Chaoran Xu, Haiyang Wei, Wenqi Fan, Tianzhong Li

**Affiliations:** Laboratory of Fruit Cell and Molecular Breeding, China Agricultural University, Beijing, 100193 China

**Keywords:** Plant breeding, Biotic

## Abstract

*Alternaria* leaf spot in apple (*Malus* x *domestica*), caused by the fungal pathogen *Alternaria alternata* f. sp. *mali* (also called *A. mali*), is a devastating disease resulting in substantial economic losses. We previously established that the resistance (R) protein MdRNL2, containing a coiled-coil, nucleotide-binding, and leucine-rich repeat (CC_R_-NB-LRR) domain, interacts with another CC_R_-NB-LRR protein, MdRNL6, to form a MdRNL2–MdRNL6 complex that confers resistance to *A. mali*. Here, to investigate the function of the MdRNL2–MdRNL6 complex, we identified two novel pathogenesis-related (PR) proteins, MdPR10-1 and MdPR10-2, that interact with MdRNL2. Yeast two-hybrid (Y2H) assays and bimolecular fluorescence complementation (BiFC) assays confirmed that MdPR10-1 and MdPR10-2 interact with MdRNL2 and MdRNL6 at the leucine-rich repeat domain. Transient expression assays demonstrated that accumulation of MdPR10-1 and MdPR10-2 enhanced the resistance of apple to four strains of *A. mali* that we tested: ALT1, GBYB2, BXSB5, and BXSB7. In vitro antifungal activity assays demonstrated that both the proteins contribute to *Alternaria* leaf spot resistance by inhibiting fungal growth. Our data provide evidence for a novel regulatory mechanism in which MdRNL2 and MdRNL6 interact with MdPR10-1 and MdPR10-2 to inhibit fungal growth, thereby contributing to *Alternaria* leaf spot resistance in apple. The identification of these two novel PR proteins will facilitate breeding for fungal disease resistance in apple.

## Introduction

Apple *Alternaria* leaf spot caused by the fungal pathogen *Alternaria alternata* f. sp. *mali* (also called *A. mali*) is a devastating and economically major disease that causes severe early defoliation and weakens tree vigor, leading to reduced apple production^1^. In infected trees, brown or black leaf spots appear on the leaflets; these rapidly spread and fuse into brown or black necrotic lesions, causing leaves to fall off^2,3^. Fruits can also be infected, resulting in brown or black sunken lesions that do not increase in size over time^2,3^. Current management methods for *Alternaria* leaf spot primarily involve traditional chemical control agents, which are expensive and damaging to the environment. Identifying the molecular mechanisms involved in *Alternaria* leaf spot resistance in apple would set the stage for selecting for, or enhancing, these beneficial mechanisms^4^.

When fungi attack apple leaves, they typically produce specific toxins and effectors, which cause the accumulation of hydrogen peroxide (H_2_O_2_); the generation of reactive oxygen species and hormones, such as jasmonic acid, salicylic acid and ethylene, and the activation of defense-related genes, ultimately leading to cell death^5,6^. Plants have evolved a variety of disease resistance (*R*) proteins that mediate the recognition of pathogen effectors and activate downstream plant immunity signaling responses, enabling them to overcome fungal pathogen attack^7^. Fungal effectors that overcome PAMP-triggered immunity (PTI) are recognized by one of the *R* proteins predicted to encode intracellular proteins containing nucleotide-binding (NB) and leucine-rich repeat (LRR) domains, and the LRR domain is a conserved feature of many R proteins^8^. In pepper (*Capsicum annuum*), pathogenesis-related protein 10 (PR10) forms a complex with leucine-rich repeat protein 1 (LRR1), and silencing of PR10/LRR1 compromises resistance to avirulent *Xanthomonas campestris* pv *vesicatoria* infection; in contrast, overexpression of heterologous PR10 confers enhanced resistance in *Arabidopsis*^9^. Thus, the LRR1–PR10 complex is responsible for cell death-mediated defense signaling, and this role is strengthened by interaction with LRR1^9^.

Pathogenesis-related (PR) genes are induced by pathogen infection, and their expression is associated with enhanced resistance to pathogens; thus, PR proteins may act as antimicrobial agents in defense signaling processes, such as cell wall hydrolysis and contact toxicity^10^. Based on their primary structure and biological activity, PR proteins have been grouped into 17 families^10,11^. According to sequence analysis, among the 17 families, the PR10 family is similar to a major birch (*Betula alba*) pollen allergen, Bet v 1, and has functions in antimicrobial activity, developmental processes, and secondary metabolism^12–17^. Several *PR10* family genes have been identified from diverse plant species, including birch (*Betula alba*), asparagus (*Asparagus officinalis*), parsley (*Petroselinum crispum*), pea (*Pisum sativum*), common bean (*Phaseolus vulgaris*), potato (*Solanum tuberosum*), sorghum (*Sorghum bicolor*), rice (*Oryza sativa*), pepper, and apple^15,17–24^. Rice blast lesion mimic mutants *Os-PR10a* and *Os-PR10b* have high expression levels, and mutants show a spontaneous cell death phenotype^25,26^. Mal d 1 (PR10), an 18-kDa intracellular PR protein, is the major apple allergen in Central and Northern Europe. The expression of *PR10* is induced by the microbial attack, fungal elicitors, and wounding stress in monocot asparagus, parsley, and bean^22–24^; however, the specific functions of PR10 in plant immunity signaling remain unclear^10^.

“Hanfu”, an apple cultivar commonly grown in China, is highly resistant to the *A. mali* strain ALT1. We previously established that HF plants inoculated with ALT1 accumulate MdRNL2 and are resistant to *A. mali*^25^. However, the role of MdRNL2 resistance in *A. mali* remains unclear. To further understand the MdRNL2 function, we performed semi–in vivo pull-down assays and liquid chromatography–mass spectrometry (LC–MS). In addition to proteins pulled down with an empty vector, there were 12 proteins in the LC–MS results, which were classified into three groups: plant defense, protein biosynthesis, and energy metabolism. From the LC–MS results, we established that the R protein MdRNL2 interacts with another R protein, MdRNL6, to form a complex through the NB-ARC domains of the proteins and that this interaction is necessary for resistance to *A. mali* (unpublished data). In addition to MdRNL6, we identified two PR10 members, which we named MdPR10-1 (major allergen Mal d 1-like; NCBI: XM_008352950.2) (Supplemental Figure 1A) and MdPR10-2 (major allergen Mal d 1; NCBI: NM_001294363.1) (Supplemental Figure 1B). To clarify the mechanism by which the MdRNL2–MdRNL6 complex inhibits ALT1 infection, we investigated the relationship among MdRNL2–MdRNL6, MdPR10-1, and MdPR10-2, as well as the roles of MdPR10-1 and MdPR10-2 in *Alternaria* leaf spot resistance. The identification of these two previously unknown proteins related to pathogenesis provides important insight that should facilitate breeding for resistance to fungal disease in apple.

## Results

### Identification of two novel pathogenesis-related proteins in apple: MdPR10-1 and MdPR10-2

Protein–protein interaction and LC–MS analyses identified two PR proteins, MdPR10-1 and MdPR10-2, that interact with MdRNL2 (Supplemental Figure 1). We cloned the cDNA sequences corresponding to *MdPR10-1* and *MdPR10-2* from HF and searched for them in the NCBI BLASTn database (https://blast.ncbi.nlm.nih.gov/). *MdPR10-1* is located on chromosome 13, and *MdPR10-2* is located on chromosome 16 (Supplemental Figure 2). Our phylogenetic analysis showed that *MdPR10-1* and *MdPR10-2* are closely related to *PR10* gene sequences from other species. *MdPR10-1* and *MdPR10-2* are most closely related to *PR10* gene sequences from pear (*Pyrus bretschneideri*), peach (*Prunus persica*), and sweet cherry (*Prunus avium*), revealing a high degree of conservation in these species. In contrast, pathogenesis-related protein 1 from *Malus* × *domestica* (MdPR1), which was used as an outgroup gene for phylogenetic analysis, was not closely related to these genes (Supplemental Figure 3). After deducing the protein sequences of MdPR10-1 and MdPR10-2 from the nucleotide sequences, we found that MdPR10-1 and MdPR10-2 are each 159 amino acids long (Supplemental Figure 4A) and share the GXGGXGXXK consensus sequence and the Bet v 1 motif, as determined by BLAST analysis (https://www.ncbi.nlm.nih.gov/Structure/cdd/wrpsb.cgi) and SnapGene software using the PR10 signature as a query (Supplemental Figure 4A, B).

### MdPR10-1 and MdPR10-2 physically interact with MdRNL2–MdRNL6

**Fig. 1:**
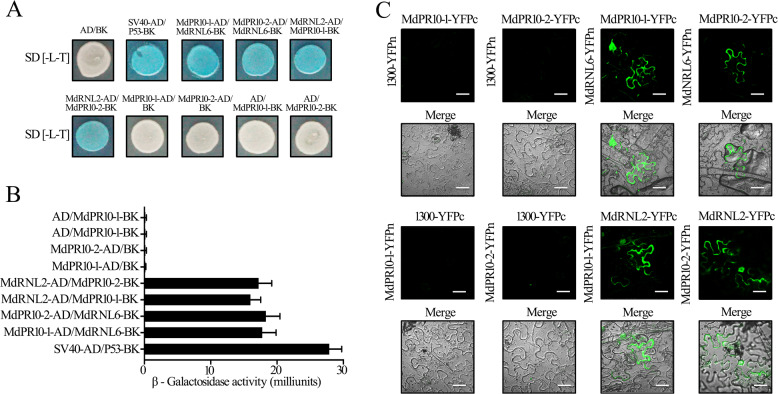
MdPR10-1 and MdPR10-2 physically interact with MdRNL2–MdRNL6. **A** Yeast two-hybrid (Y2H) analysis showing that both MdPR10-1 and MdPR10-2 interact with MdRNL2 and MdRNL6. Coexpression of SV40-AD/BD-P53 was used as a positive control. As negative controls, MdPR10-1-AD/BK, MdPR10-2-AD/BK, AD/MdPR10-1-BK, AD/MdPR10-2-BK, and AD/BD were coexpressed. **B** β-Galactosidase activity in Y2H assays. The error bars show the standard error of the mean (SEM) values (*n* = 3 biological replicates). **P < 0.01 (Student’s *t* test). **C** BiFC assay of the interactions between MdPR10-1 and MdRNL2–MdRNL6 and between MdPR10-2 and MdRNL2–MdRNL6. MdRNL2-YFPc/MdPR10-1-YFPn, MdRNL2-YFPc/MdPR10-2-YFPn, MdPR10-1-YFPc/MdRNL6-YFPn, and MdPR10-2-YFPc/MdRNL6-YFPn were transiently expressed in *N. benthamiana* epidermal cells. 1300-YFPc/MdPR10-1-YFPn, 1300-YFPc/MdPR10-2-YFPn, MdPR10-1-YFPc/1300-YFPn, and MdPR10-2-YFPc/1300-YFPn were used as negative controls. Scale bars = 50 μm

*MdPR10-1* and *MdPR10-2* were highly induced in HF by ALT1 inoculation, whereas the outgroup gene *MdPR1* was not induced (Supplemental Figure 5). Next, we tested for interactions between MdRNL2–MdRNL6 and the two identified PR proteins using yeast two-hybrid (Y2H) assays. We cloned the *MdPR10-1* and *MdPR10-2* coding sequences into pGADT7 and pGBKT7 (yielding MdPR10-1-AD, MdPR10-2-AD, MdPR10-1-BK, and MdPR10-2-BK) and cotransfected the combinations MdRNL2-AD/MdPR10-1-BK, MdRNL2-AD/MdPR10-2-BK, MdPR10-1-AD/MdRNL6-BK, and MdPR10-2-AD/MdRNL6-BK into the yeast strain AH109 (Fig. 1A). Colonies containing any of the four combinations grew on synthetic dropout nutrient medium and were stained with X-α-gal, suggesting that MdPR10-1 and MdPR10-2 each interact with both MdRNL2 and MdRNL6 (Figs. 1A, B).

To verify the interaction of MdPR10-1 and MdPR10-2 with MdRNL2–MdRNL6 in plant cells, we conducted a bimolecular fluorescence complementation (BiFC) assay in *Nicotiana benthamiana*. The combinations MdPR10-1-YFPc/1300-YFPn, MdPR10-2-YFPc/1300-YFPn, MdPR10-1-YFPc/MdRNL6-YFPn, MdPR10-2-YFPc/MdRNL6-YFPn, 1300-YFPc/MdPR10-1-YFPn, 1300-YFPc/MdPR10-2-YFPn, MdRNL2-YFPc/MdPR10-1-YFPn, and MdRNL2-YFPc/MdPR10-2-YFPn were each transiently expressed in *N. benthamiana* leaves using *Agrobacterium tumefaciens* transfection (Fig. 1C). As expected, epidermal cells expressing MdPR10-1-YFPc/MdRNL6-YFPn, MdPR10-2-YFPc/MdRNL6-YFPn, MdRNL2-YFPc/MdPR10-1-YFPn, and MdRNL2-YFPc/MdPR10-2-YFPn showed strong fluorescence signals, demonstrating that MdPR10-1 and MdPR10-2 interact with both MdRNL2 and MdRNL6 in vivo (Fig. 1C). These results suggest that MdPR10-1 and MdPR10-2 physically bind to MdRNL2–MdRNL6 in apple.

### MdPR10-1 and MdPR10-2 physically bind to MdRNL2–MdRNL6 at the LRR domain

**Fig. 2:**
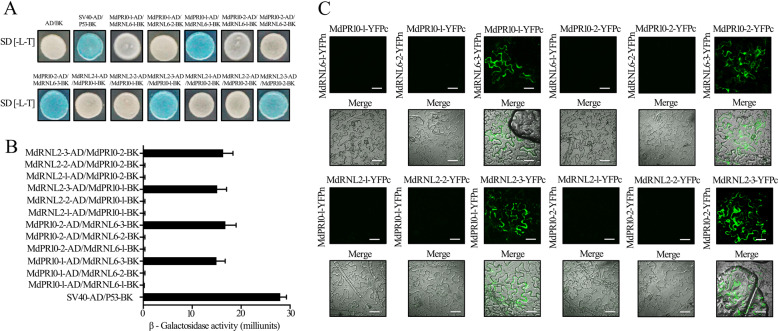
MdPR10-1 and MdPR10-2 physically interact with MdRNL2–MdRNL6 at the LRR domain. **A** Y2H analysis showing the interactions between MdPR10-1 and MdRNL2-3, MdPR10-2 and MdRNL2-3, MdPR10-1 and MdRNL6-3, and MdPR10-2 and MdRNL6-3. **B** β-Galactosidase activity in Y2H assays. The error bars show the standard error of the mean (SEM) values (*n* = 3 biological replicates). **P < 0.01 (Student’s *t* test). **C** BiFC assay of the interactions between MdPR10-1 and MdRNL2-3, MdPR10-2 and MdRNL2-3, MdPR10-1 and MdRNL6-3, and MdPR10-2 and MdRNL6-3 when transiently expressed in *Nicotiana benthamiana* epidermal cells. Scale bars = 50 μm

To investigate whether the interactions of MdPR10-1 and MdPR10-2 with MdRNL2 and MdRNL6 involve direct physical binding and to identify the protein domains involved, we analyzed the pairwise interactions between various truncated versions of MdRNL2–MdRNL2-1 (CC_R_ domain of MdRNL2), MdRNL2-2 (NB-ARC domain of MdRNL2), MdRNL2-3 (LRR domain of MdRNL2), MdRNL6-1 (CC_R_ domain of MdRNL6), MdRNL6-2 (NB-ARC domain of MdRNL6) or MdRNL6-3 (LRR domain of MdRNL6) and MdPR10-1 or MdPR10-2 using the Y2H system. Coexpression of MdPR10-1-AD/MdRNL6-1-BK, MdPR10-1-AD/MdRNL6-2-BK, MdPR10-1-AD/MdRNL6-3-BK, MdPR10-2-AD/MdRNL6-1-BK, MdPR10-2-AD/MdRNL6-2-BK, MdPR10-2-AD/MdRNL6-3-BK, MdRNL2-1-AD/MdPR10-1-BK, MdRNL2-2-AD/MdPR10-1-BK, MdRNL2-3-AD/MdPR10-1-BK, MdRNL2-1-AD/MdPR10-2-BK, MdRNL2-2-AD/MdPR10-2-BK, and MdRNL2-3-AD/MdPR10-2-BK allowed growth on the selective media (Fig. 2A), and staining with X-α-gal revealed that MdPR10-1 and MdPR10-2 interacted with MdRNL2-3 and MdRNL6-3. These data indicate that the LRR domains of MdRNL2 and of MdRNL6 interact with MdPR10-1 and MdPR10-2 (Figs. 2A, B). We validated this finding through a BiFC assay. The combinations MdPR10-1-YFPc/MdRNL6-3-YFPn, MdPR10-2-YFPc/MdRNL6-3-YFPn, MdRNL2-3-YFPc/MdPR10-1-YFPn, and MdRNL2-3-YFPc/MdPR10-2-YFPn resulted in strong fluorescence signals in the *N. benthamiana* epidermal cell cytoplasm (Fig. 2C). Thus, MdPR10-1 and MdPR10-2 appear to bind to the LRR domains of MdRNL2 and MdRNL6.

### MdPR10-1 and MdPR10-2 are both required for ALT1 resistance

**Fig. 3:**
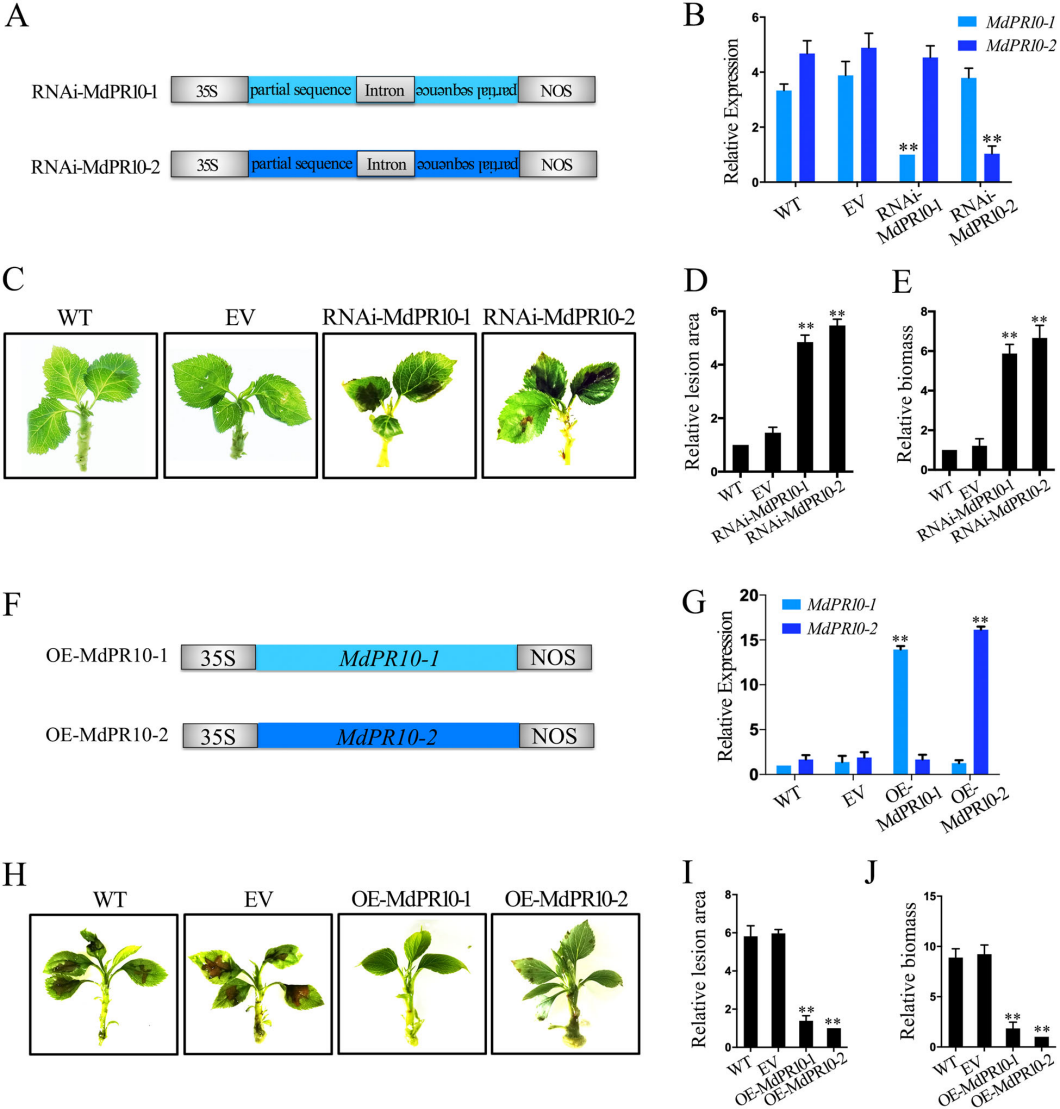
MdPR10-1 and MdPR10-2 participate in resistance to ALT1 in apple. **A** Schematic of the constructs used for silencing of *MdPR10-1* (RNAi-MdPR10-1) and *MdPR10-2* (RNAi-MdPR10-2). **B**–**D** MdPR10-1 and MdPR10-2 transcript contents revealed by RT-qPCR **B**, infection symptoms **C**, relative lesion areas **D**, and relative biomass of *A. mail* **E** in the WT and empty vector (EV)-, RNAi-MdPR10-1-, and RNAi-MdPR10-2-infiltrated Hanfu (HF) plants at 48 hpi with ALT1. **F** Schematic of constructs used for overexpression of *MdPR10-1* (OE-MdPR10-1) and *MdPR10-2* (OE-MdPR10-2). **G**–**J** MdPR10-1 and MdPR10-2 contents revealed by RT-qPCR **G**, infection symptoms **H**, relative lesion areas **I**, and relative biomass of *A. mail* **J** in the WT and in EV-, OE-MdPR10-1-, and OE-MdPR10-2-infiltrated NGR196 plants at 48 hpi. The spore inoculum concentration was 2 × 10^5^ CFU/mL. Error bars in **B**, **D**, **E**, **G**, **I**, and **J** = SDs; **P < 0.01 (Student’s *t* test). Similar results were obtained in three independent biological replicates

We next investigated the functional relationship between the MdPR10-1 and MdPR10-2 during ALT1 infection in apple. We constructed a binary vector (pFGC5941) containing partial sequences of MdPR10-1 and MdPR10-2 and their corresponding reverse complementary sequences to form hairpin structures (RNAi-MdPR10-1 and RNAi-MdPR10-2). This produced small interfering RNAs that specifically silenced MdPR10-1 and MdPR10-2 when transiently expressed in HF via agroinfiltration (Fig. 3A). Four days after transformation, the expression levels of MdPR10-1 and MdPR10-2 were downregulated in RNAi-MdPR10-1 and RNAi-MdPR10-2 HF seedlings (Fig. 3B). Forty-eight hours postinoculation (hpi) with ALT1, the average percentage of leaf area affected by lesions was significantly higher in RNAi-MdPR10-1 and RNAi-MdPR10-2 HF seedlings than in wild-type controls (WT; uninfiltrated HF seedlings) or empty vector controls (EV; HF seedlings agroinfiltrated with empty vector), demonstrating that resistance to ALT1 infection was suppressed (Figs. 3C, D). Reverse transcription-quantitative PCR (RT-qPCR) analysis showed that the *A. mali* biomass was increased in the RNAi-MdPR10-1 and RNAi-MdPR10-2 HF lines, explaining their loss of resistance (Fig. 3E). The gene-silencing screen indicated that the MdRNL2–MdRNL6 complex is required for MdPR10-1- and MdPR10-2-mediated resistance to ALT1.

In contrast, transformation of the full-length *MdPR10-1* or *MdPR10-2* transcript into the susceptible apple cultivar NGR196 via *A. tumefaciens* infiltration (yielding OE-MdPR10-1 or OE-MdPR10-2 NGR196 seedlings, respectively) (Fig. 3F) resulted in increased expression of *MdPR10-1* and *MdPR10-2* 4 d after infiltration (Fig. 3G). At 48 hpi with ALT1, the average percentage of leaf area affected by lesions was lower in OE-MdPR10-1 and OE-MdPR10-2 NGR196 plants than in control WT and EV-infiltrated NGR196 plants, indicating that their resistance to *Alternaria* leaf spot was enhanced (Figs. 3H, I). RT-qPCR analysis showed that *AMT1* expression was decreased in OE-MdPR10-1 and OE-MdPR10-2 NGR196 lines (Fig. 3J), possibly explaining why they developed resistance. These data demonstrate that the accumulation of MdPR10-1 and MdPR10-2 enhances ALT1 resistance in the susceptible NGR196 cultivar.

### MdPR10-1 and MdPR10-2 inhibit ALT1 growth in vitro

**Fig. 4:**
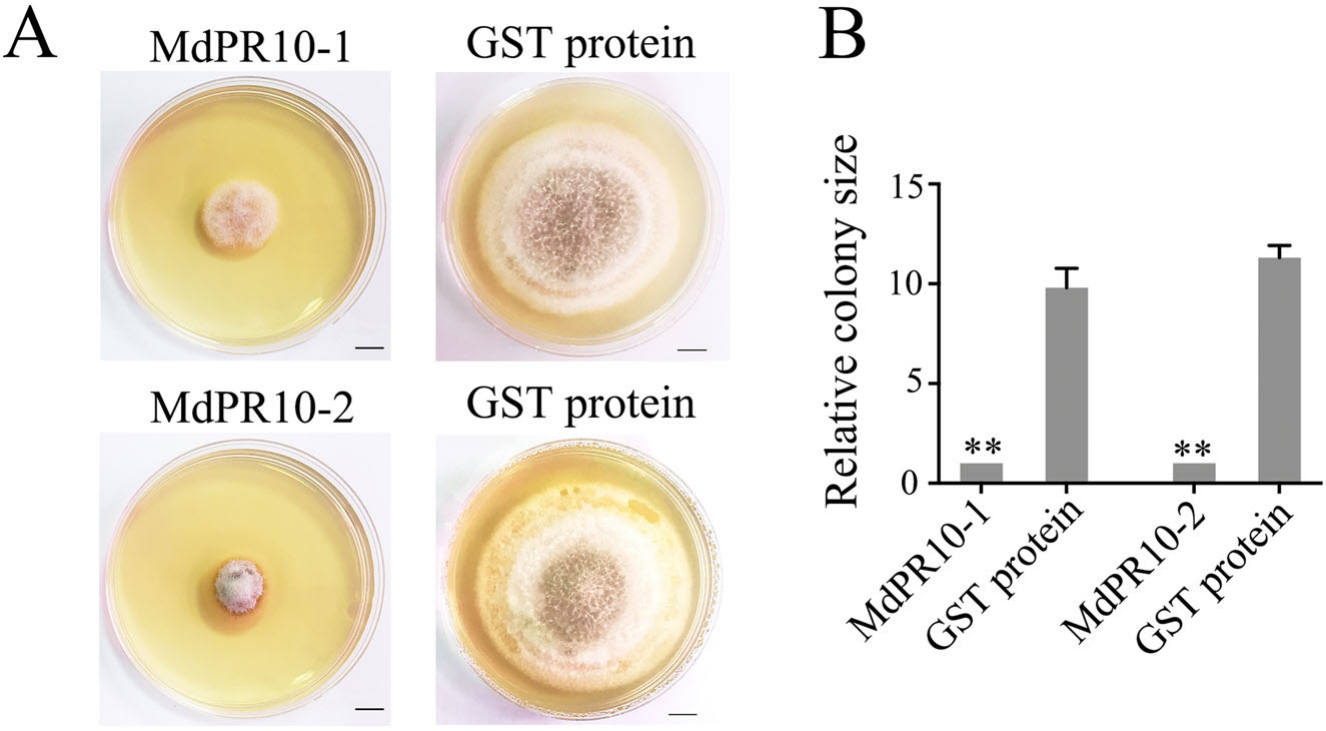
Recombinant MdPR10-1 and MdPR10-2 proteins have anti-ALT1 activity in vitro. **A** Growth inhibition of ALT1 treated with MdPR10-1 and MdPR10-2 proteins. Left: 20 μL of ALT1 sporangial suspension + 60 μL of 500 μg/μL purified MdPR10-1/MdPR10-2 proteins. Right: 20 μL of ALT1 sporangial suspension + 60 μL of 500 μg/μL of purified GST proteins. **B** Area of ALT1 mycelium growth in PDA, as determined using ImageJ. The spore suspension concentration was 2 × 10^5^ CFU/mL. The data were collected on day 6 in PDA. Scale bars = 1 cm. Student’s *t* test: **P < 0.01

Next, we clarified the mechanism by which MdPR10-1 and MdPR10-2 contribute to ALT1 resistance and tested whether MdPR10-1 and MdPR10-2 have a direct effect on fungal growth. We purified the recombinant MdPR10-1 and MdPR10-2 proteins with glutathione *S*-transferase (GST) resin and detected them on a 12% sodium dodecyl sulfate-polyacrylamide gel electrophoresis (SDS-PAGE) gel (Supplemental Figure 6). An in vitro antifungal activity assay using these purified proteins confirmed that ALT1 growth was strongly inhibited in the groups treated with MdPR10-1 or MdPR10-2 compared with the untreated control group (Fig. 4A). A quantitative analysis of the antifungal activity of MdPR10-1 and MdPR10-2 indicated that the area of ALT1 mycelium growth in potato dextrose agar (PDA) differed greatly between cultures treated with MdPR10-1 and MdPR10-2 and the negative controls (Fig. 4B), suggesting that MdPR10-1 and MdPR10-2 contribute to ALT1 resistance by inhibiting fungal growth.

### MdPR10-1 and MdPR10-2 are both required for GBYB2, BXSB5, and BXSB7 resistance

**Fig. 5:**
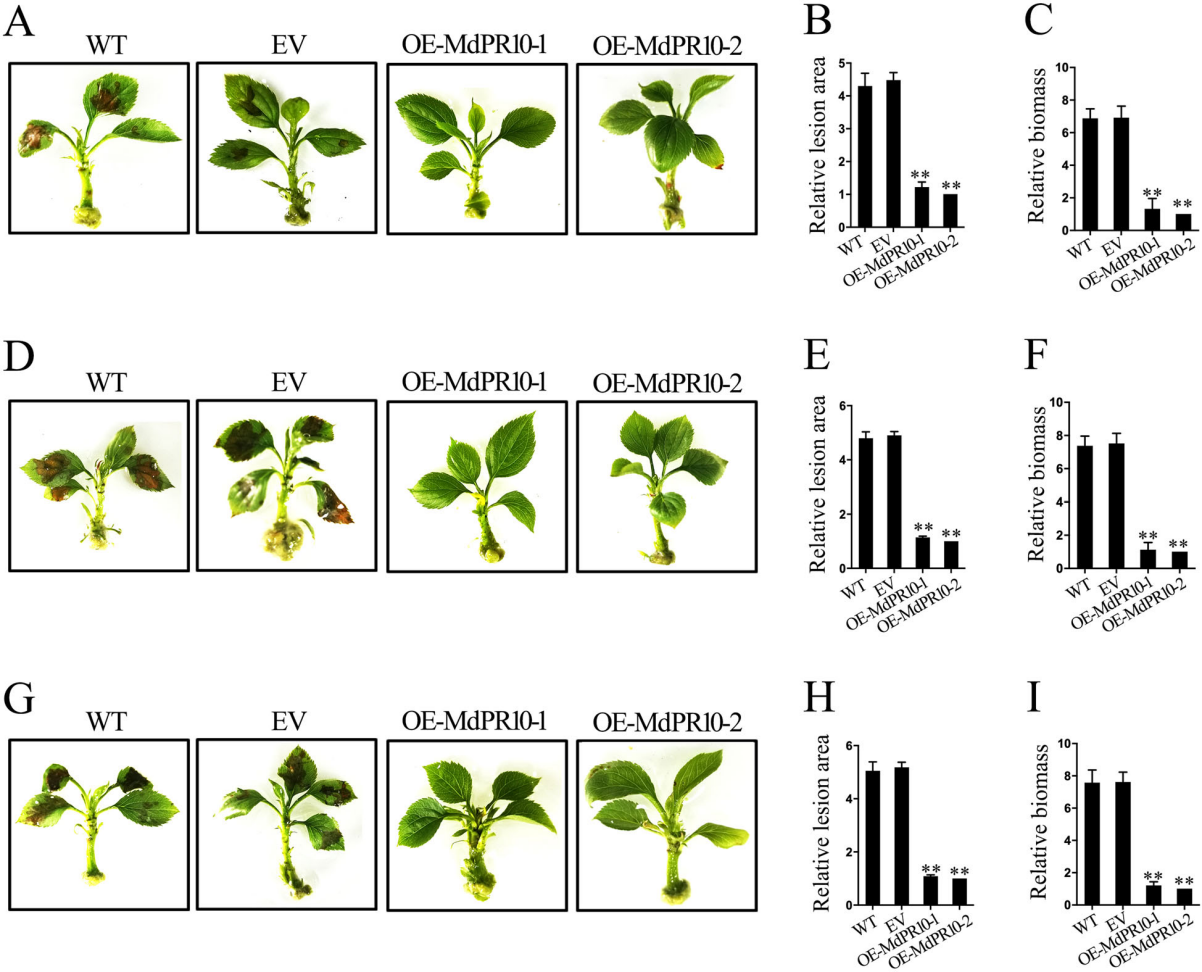
MdPR10-1 and MdPR10-2 participate in resistance to GBYB2, BXSB5, and BXSB7 in apple. **A** Infection symptoms of WT and EV-, OE-MdPR10-1-, and OE-MdPR10-2-infiltrated NGR196 plants at 48 hpi with GBYB2. **B**, **C** Relative lesion areas **B** and relative biomass of *A. mail* **C** in the leaves of these plants. **D**–**F** Infection symptoms **D**, relative lesion areas **E**, and relative biomass of *A. mail* **F** in the leaves of WT and EV-, OE-MdPR10-1-, and OE-MdPR10-2-infiltrated NGR196 plants at 48 hpi with BXSB5. **G**–**I** Infection symptoms **G**, relative lesion areas **H**, and relative biomass of *A. mail* (I) in WT and EV-, OE-MdPR10-1-, and OE-MdPR10-2-infiltrated NGR196 plants at 48 hpi with BXSB7 plants. Throughout, the spore inoculum concentrations were 2 × 10^5^ CFU/mL. Error bars = SDs; **P < 0.01 (Student’s *t* test). Similar results were obtained from three independent biological replicates

To further explore the contributions of MdPR10-1 and MdPR10-2 to *Alternaria* leaf spot resistance, we studied three other strains of *A. mali*: GBYB2, BXSB5, and BXSB7. We transformed the full-length transcripts of MdPR10-1 and MdPR10-2 into the susceptible apple cultivar NGR196 via *A. tumefaciens* infiltration (Fig. 3F). Four days after infiltration, we observed increased *MdPR10-1* and *MdPR10-2* expression levels in OE-MdPR10-1 and OE-MdPR10-2 NGR196 seedlings, respectively (Supplemental Figure 7). At 48 hpi with GBYB2, BXSB5, or BXSB7, the average percentage of leaf area affected by lesions was lower in OE-MdPR10-1 and OE-MdPR10-2 plants than in control WT or EV-infiltrated plants, indicating that their resistance to *Alternaria* leaf spot was enhanced (Fig. 5). RT-qPCR analysis showed that *AMT1* expression was decreased in OE-MdPR10-1 and OE-MdPR10-2 NGR196 lines inoculated with GBYB2, BXSB5, or BXSB7 (Fig. 5), possibly explaining their development of resistance. These data demonstrate that the accumulation of MdPR10-1 and MdPR10-2 enhances resistance to GBYB2, BXSB5, and BXSB7 in the NGR196 cultivar.

### MdPR10-1 and MdPR10-2 inhibit GBYB2, BXSB5, and BXSB7 growth in vitro

**Fig. 6:**
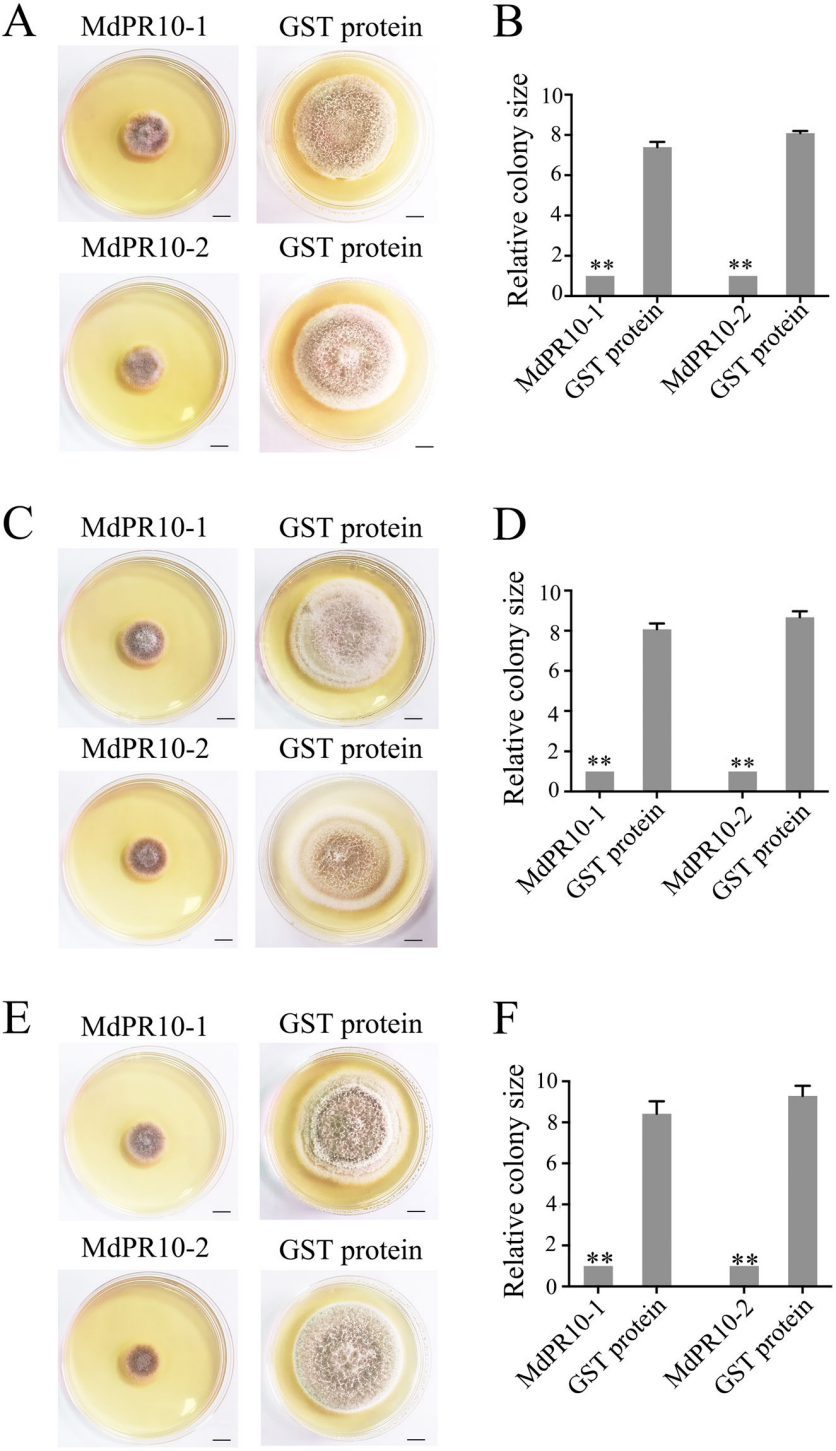
Recombinant MdPR10-1 and MdPR10-2 exert anti-GBYB2/BXSB5/BXSB7 activity in vitro. **A** Growth inhibition of GBYB2 treated with the MdPR10-1 and MdPR10-2 proteins. Left: 20 μL of GBYB2 sporangial suspension + 60 μL of 500 μg/μL of purified MdPR10-1/MdPR10-2 proteins. Right: 20 μL of GBYB2 sporangial suspension + 60 μL of 500 μg/μL of purified GST proteins. **B** Area of GBYB2 mycelium growth in PDA, as determined using ImageJ. **C** Growth inhibition of BXSB5 treated with the MdPR10-1 and MdPR10-2 proteins. Left: 20 μL of BXSB5 sporangial suspension + 60 μL of 500 μg/μL purified MdPR10-1/MdPR10-2 proteins. Right: 20 μL of BXSB5 sporangial suspension + 60 μL of 500 μg/μL purified GST proteins. **D** Area of BXSB5 mycelium growth in PDA, as determined with ImageJ. **E** Growth inhibition of BXSB7 treated with the MdPR10-1 and MdPR10-2 proteins. Left: 20 μL of BXSB7 sporangial suspension + 60 μL of 500 μg/μL purified MdPR10-1/MdPR10-2 proteins. Right: 20 μL of BXSB7 sporangial suspension + 60 μL of 500 μg/μL purified GST proteins. **F** Area of BXSB7 mycelium growth in PDA, as determined with ImageJ. Throughout, the spore suspension concentration was 2 × 10^5^ CFU/mL. The data were collected on day 6 in PDA. Scale bars = 1 cm. Student’s *t*-test: **P < 0.01

In vitro antifungal activity assays confirmed that GBYB2, BXSB5, and BXSB7 growth was strongly inhibited in the groups treated with the recombinant proteins MdPR10-1 and MdPR10-2 compared with the negative control group (Figs. 6A, C, and E). In a quantitative analysis of antifungal activity, the area of mycelium growth in PDA differed greatly in samples treated with purified MdPR10-1 or MdPR10-2 compared with samples treated with the negative control (Figs. 6B, D, and F). These observations suggest that MdPR10-1 and MdPR10-2 contribute to GBYB2, BXSB5, and BXSB7 resistance by inhibiting fungal growth.

## Discussion

Several *PR-10* genes have been isolated in various species with distinct expression patterns in response to stress conditions^26,27^. The first reported *PR10* gene from parsley established the PR-10 class of PR proteins^22,28^. Shortly afterward, according to their sequence homology with PR-10 proteins, other proteins found in birch^17^, celery^29^, apple^30^, and other fruits and vegetables were also included in the PR-10 class, most of which were induced by pathogens^31^. In this study, we demonstrate that apple pathogenesis-related protein 10 is pivotal for apple defense and responses against fungal attack. In apple, the MdPR10-1 and MdPR10-2 genes have been identified, and the expression of *MdPR10-1* and *MdPR10-2* is induced by *A. mali*^4^. Although PR10 is known to have ribonuclease and antimicrobial activity in plants, little is known about the specific functions of PR10 in apple^13,15^. In our study, transient expression assays demonstrated that accumulation of MdPR10-1 and MdPR10-2 enhanced resistance to ALT1, GBYB2, BXSB5, and BXSB7 in apple (Fig. 3 and Fig. 5). Using in vitro antifungal activity assays, we established that MdPR10-1 and MdPR10-2 contributed to *Alternaria* leaf spot resistance by inhibiting fungal growth (Fig. 4 and Fig. 6).

In *N. benthamiana* (tobacco), overexpression of PR10 alone does not induce a cell death response in leaves^32,33^. Additionally, the PR10 family is well characterized at the structural level^34^. Until now, there has been no relative experimental evidence of any physical interaction between PR10 and other proteins in apple. The Bet v 1 fold domain in PR10 is responsible for its interaction with several types of ligands, such as cytokinins, brassinosteroids, and flavonoids, and might also be involved in its binding to LRR1; for example, CaPR10 forms a complex with LRR1 in pepper, which is resistant to bacterial infection^9,35–38^. In our study, Y2H and BiFC assays confirmed that both the MdPR10-1 and MdPR10-2 interact with MdRNL2–MdRNL6 at the LRR domain (Fig. 1 and Fig. 2). We established that the LRR domains of MdRNL2–MdRNL6 physically interact with MdPR10-1 and MdPR10-2, enhancing PR10-triggered defense responses in apple (Fig. 1 and Fig. 2).

*Alternaria* leaf blotch caused by *Alternaria spp*. leads to significant losses in apple production. When *A. mali* attacks apple leaves, the leaves first develop brown or black blotches surrounded by a dark margin; then, the leaves turn yellow and abscise prematurely from the canopy^39^. Leaf blotch mostly affects high-value and very popular cultivars, such as the ‘Fuji’, ‘Royal Gala’, ‘Red Delicious’, and ‘Pink Lady’ cultivars^39^. *A. mali*, referred to as the apple *Alternaria* leaf blotch pathotype, has been reported in Japan^40^, China^41^, Korea^42^, the United States^43^, Russia^44^, Yugoslavia^45^, Iran^46^, Turkey^47^, and Brazil^48^. Few studies have investigated *Alternaria* leaf blotch control and resistance genes. Our data provide detailed evidence for a novel regulatory mechanism in which MdRNL2–MdRNL6 interact with MdPR10-1 and MdPR10-2 to inhibit fungal growth, thereby contributing to *Alternaria* leaf spot resistance in apple (Supplemental Figure 8). These data warrant an investigation into the role of PR10 in defense responses in apple and will facilitate breeding for fungal disease resistance in this species.

## Materials and methods

### Plant materials

Tissue culture seedlings of different apples (*Malus domestica* cv. ‘Hanfu’, HF; *Malus domestica* cv. NGR196) were planted on Murashige and Skoog (MS) medium containing 0.6 mg/L 6-benzylaminopurine (6-BA) and 0.15 mg/L 1-naphthylacetic acid (NAA). The temperature of the culture room was controlled at 24 ± 1 °C, and the photoperiod was set as 16 h/8 h light/dark under fluorescent lamps (Philips TL5 28 W/865). Every four weeks, the seedlings were transferred to a fresh medium, and the plants were used for fungal infection and *Agrobacterium tumefaciens* infiltration experiments.

### Fungal infection assay

Four strains of *A*. *mali*, ALT1, GBYB2, BXSB5, and BXSB7, were cultured on potato dextrose agar (PDA) medium in the dark, and the temperature was controlled at 25 °C. After 6 days of growth, the spores were diluted in double-distilled water, and the final concentration was quantified as 2 × 10^5^ CFU/mL via observation under a microscope (Olympus CX31). Usually, 4 d after *A. tumefaciens* infiltration, spore suspensions of ALT1, GBYB2, BXSB5, or BXSB7 pathogens were inoculated on 4-week-old apple leaves^4^.

The lesion size was measured as described previously^49^. First, we removed leaves from more than 10 inoculated *A*. *mali* apple plantlets (approximately 30–40 leaves) at 48 hpi, and images of the leaves were taken with a scale bar. The lesion sizes were then calculated using ImageJ software. The results for three independent biological replicates were obtained.

Calculation of the relative DNA content of ALT1/GBYB2/BXSB5/BXSB7 in apple was performed as described previously^49,50^. After 48 h of inoculation with *A*. *mali*, the infected apple leaves were collected, and four leaves from different infected seedlings were regarded as one sample. DNA of apple leaves was extracted and purified by using a DNeasy Plant Mini Kit (Qiagen, 69104). SuperReal PreMix Plus (SYBR Green) (Tiangen, FP205) was used for the following real-time PCR assay, and the relative DNA abundance was calculated by the 2^−ΔΔ*C*T^ method^51^. The reference gene was *MdActin* (NCBI XM_008365636.2). The specific primers for *AMT1* and the reference gene are listed in Supplemental Table 2.

### Protein purification

MdPR10-1 and MdPR10-2 were inserted into the pGEX-4T-1 vector and transformed into *Trans*etta(DE3) chemically competent *Escherichia coli* cells, whose growth was then induced at 16 °C. After 16 hours, the cells were suspended in suspension buffer (10 mM Tris-HCl, pH 7.4, 30 mM NaCl). Samples of the *E. coli* suspension culture were pulverized with ultrasound for 20 minutes and then pelleted by centrifugation (12,000 *g*, 1 h, 4 °C). The collected supernatant was purified with GST resin (Life Technologies, G2879) at 4 °C for 1 hour. The supernatant was then discarded, and the remaining beads were rinsed with glutathione buffer three times before being used for further experiments.

### Yeast two-hybrid (Y2H) assays

For Y2H assays, the coding sequences of *MdRNL2*, *MdRNL2-1*, *MdRNL2-2*, *MdRNL2-3*, *MdPR10-1*, and *MdPR10-2* were fused with the pGADT7 vector (Clontech). The coding sequences of *MdRNL6*, *MdRNL6-1*, *MdRNL6-2*, *MdRNL6-3*, *MdPR10-1*, and *MdPR10-2* were fused with the pGBKT7 vector (Clontech). Different combinations of pGBKT7 and pGADT7 vectors were cotransformed into the AH109 strain, and then the transformed strains were grown on SD/–Leu–Trp medium at 30 °C for 4–5 days. For each combination, three independent experiments were carried out. The primers are shown in Supplemental Table 1.

### Bimolecular fluorescence (BiFC) assays

The coding sequences of *MdRNL2*, *MdRNL2-1*, *MdRNL2-2*, *MdRNL2-3*, *MdRNL6*, *MdRNL6-1*, *MdRNL6-2*, *MdRNL6-3*, *MdPR10-1*, and *MdPR10-2* were amplified and fused with the coding sequence of the N-terminus of YFP (YFPn) or the C-terminus of YFP (YFPc) and inserted into vectors. Various combinations of YFPn and YFPc were transiently expressed in apple leaves by *Agrobacterium tumefaciens* infiltration^52^. Four days later, YFP fluorescence in leaves was observed and imaged at wavelengths of 500–542 nm using a laser scanning confocal microscope (Olympus BX61). The cloning primers are listed in Supplemental Table 2.

### Agrobacterium tumefaciens infiltration

In overexpression experiments, full-length *MdPR10-1* and *MdPR10-2* sequences were cloned into the vector pFGC5941 (GenBank AY310901) with NcoI/BamHI restriction sites. The empty pFGC5941 vector was used as the control. Loss-of-function *MdPR10-1* and *MdPR10-2* constructs were produced by cloning their specific sequences and their partially specific reverse sequences into pFGC5941 using the NcoI/SawI and XbaI/BamHI restriction sites for *MdPR10-1* and *MdPR10-2*, respectively, to produce small interfering RNAs (siRNAs). The above vectors were transformed into GV3101 (an *A. tumefaciens* strain) by the heat shock transformation method. The cloning primers are listed in Supplemental Table 1.

Leaves from 4-week-old HF seedlings (resistant variety) and 4-week-old NGR196 seedlings (susceptible variety) were infiltrated by *A. tumefaciens* with a silencing or overexpression construct^25^. After agroinfiltration, the infiltrated seedlings were transferred to a fresh MS culture medium for 4 d to avoid wilting of the apple plantlets during *Agrobacterium tumefaciens* infiltration. After 4 days, the infiltrated apple plantlets were inoculated with a 2 × 10^5^ CFU/mL pathogenic spore suspension, and the *A*. *mali-*inoculated seedlings were returned to fresh MS culture medium for 48 h to avoid wilting of the apple plantlets during fungal inoculation^25^.

### Real-time PCR (RT-qPCR)

Total RNA of apple leaves was extracted with an EASY Spin Kit (Beijing Biomed Biotechnology Co., Ltd., China). The RNA was reverse-transcribed into cDNA using oligo-dT primers, which are listed in Supplemental Table 2. Real-time PCR analysis was performed using SuperReal PreMix Plus (SYBR Green) (Tiangen, FP205) under the following cycling procedure: 40 cycles of 95 °C for 10 s and 60 °C for 30 s (Applied Biosystems 7500). The relative RNA abundance was calculated using the 2^−ΔΔ*C*T^ method^51^, with *MdActin* as the reference gene. The specific primers for *MdPR10-1, MdPR10-2*, and the reference gene are listed in Supplemental Table 2.

### Antifungal activity in vitro

The inhibition of *A. mail* mycelium growth by MdPR10-1 or MdPR10-2 in vitro was determined via incubation in Petri dishes by culturing *A. mail* spores on PDA medium containing purified GST protein, purified MdPR10-1 protein, or purified MdPR10-2 protein for 6 days. The temperature was controlled at 25 °C, and the cultures were kept in the dark.

Mycelium quantification was performed as described previously^53^. Photographs were taken with a scale bar after 6 days of growth on PDA, and the area of mycelium growth was calculated with ImageJ (http://imagej.nih.gov/ij/). The results for three independent biological replicates were obtained.

## Supplementary information

Supplemental Materials

Supplemental Figure 1

Supplemental Figure 2

Supplemental Figure 3

Supplemental Figure 4

Supplemental Figure 5

Supplemental Figure 6

Supplemental Figure 7

Supplemental Figure 8
