# Supplementary material for: Two pathogenesis-related proteins interact with leucine-rich repeat proteins to promote Alternaria leaf spot resistance in apple
Source: Hortic Res. 2021 Oct 1;8:219. doi: 10.1038/s41438-021-00654-4 (PMC8484663; doi:10.1038/s41438-021-00654-4)
Supplement: Supplementary file 1 — Supplemental Materials [file 41438_2021_654_MOESM1_ESM.docx]

**Supplemental Material**

**Supplemental Figure 1. Schematic diagram of the LC–MS analysis with MdPR10-1 and MdPR10-2 proteins and peptide overlays.** (A) Mass spectrometry (MS) data for MdPR10-1: LC–MS analysis of MdPR10-1 protein and peptide overlays. (B) MS data for MdPR10-2: LC–MS analysis of MdPR10-2 protein and peptide overlays.


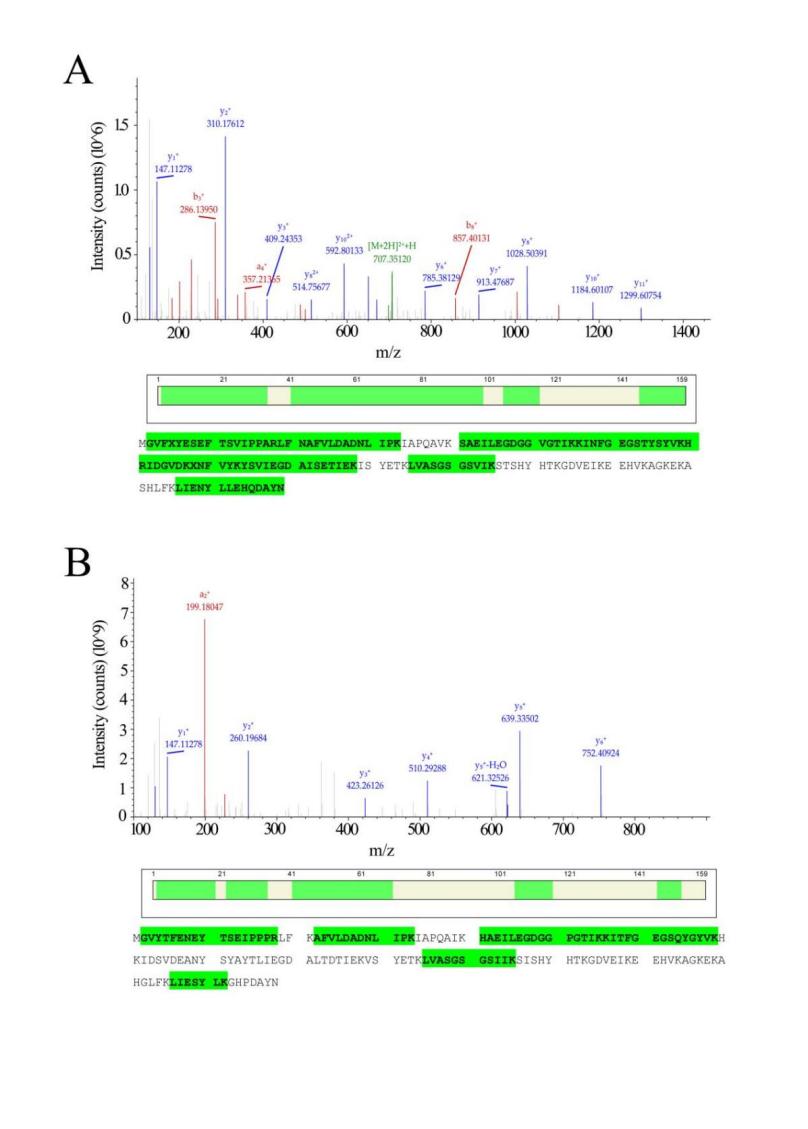


**Supplemental Figure 2. Chromosomal locations of the *MdPR10-1* and *MdPR10-2* genes.** *MdPR10-1* is located on chromosome 13, and *MdPR10-2* is located on chromosome 16.


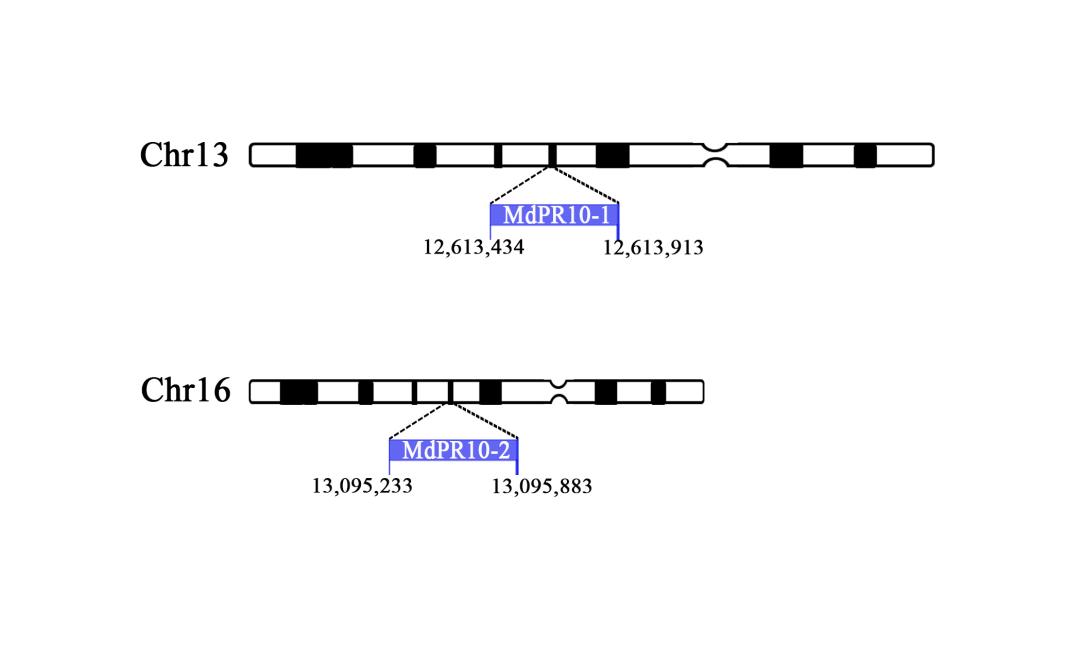


**Supplemental Figure 3. Phylogenetic analysis of *PR10* genes shows the relationship between PdPR10 and other members of the PR10 subfamily.** The phylogenetic tree was drawn using Tree View based on alignments by MEGA7. PR10 subfamily proteins used in this analysis are from *Malus domestica* (XM_008352950.2 and NM_001294363.1), *Pyrus bretschneideri* (XM_009349095.2 and XM_009349094.2), *Prunus persica* (XM_007225917.2 and XM_007223577.2), *Prunus avium* (XM_021950470.1 and XM_021950559.1), *Glycine soja* (XM_028347951.1, XM_028347719.1, and XM_028323971.1), *Glycine max* (NM_001249109.3 and NM_001251351.2), *Cajanus cajan* (XM_020379539.2), *Arachis hypogaea* (XM_025774367.1, XM_025838805.1, XM_025838816.1, and XM_025790758.1), *Capsicum annuum* (XM_016710983.1), *Gossypium raimondii* (XM_012604858.1), *Zea mays* (NC_050105.1 and XM_008656464.4), and *Hordeum vulgare* (AY220734.1), as well as PR1 (NM_001311210.1) from *Malus domestica* as an outgroup gene for phylogenetic analysis.


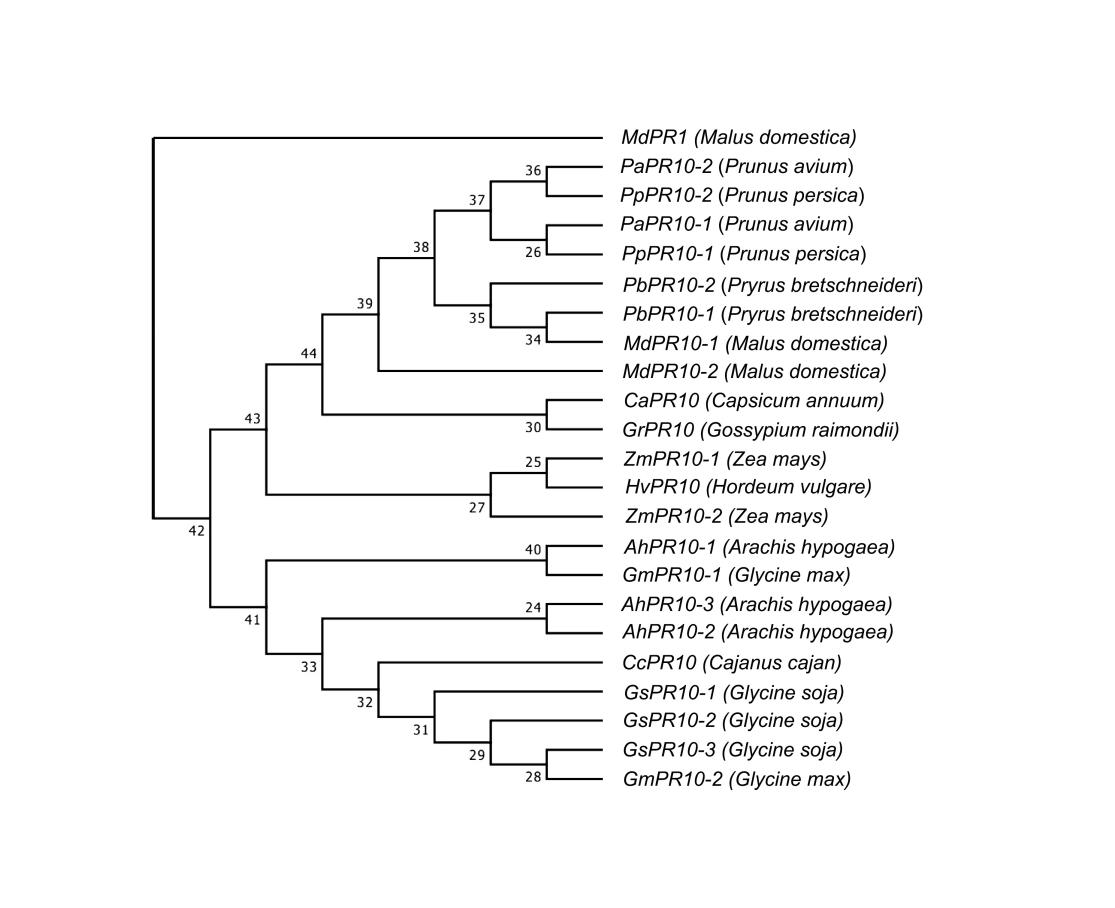


**Supplemental Figure 4. Domain analysis and sequence alignment of MdPR10-1 and MdPR10-2.** (A) MdPR10-1 and MdPR10-2 each contain a Bet_v1-like domain. (B) Sequence alignment of MdPR10-1 and MdPR10-2 proteins. The glycine-rich loop (P-loop) is framed with a rectangle.


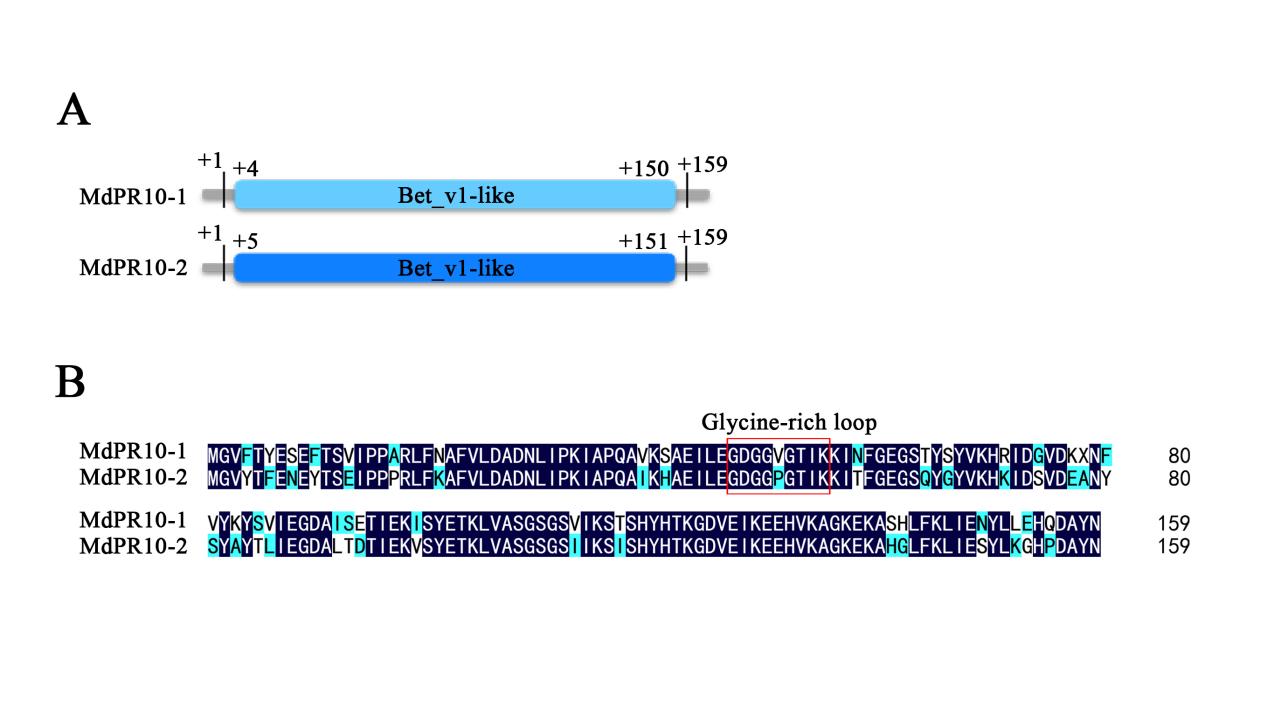


**Supplemental Figure 5. *MdPR10-1* and *MdPR10-2* expression in non-inoculated HF leaves and at 48 hpi in ALT1-inoculated HF leaves.** *MdPR10-1* and *MdPR10-2* transcript levels in HF leaves, as revealed by RT-qPCR. Error bars = SD. RT-qPCR data were calculated based on three biological and three technical replicates.


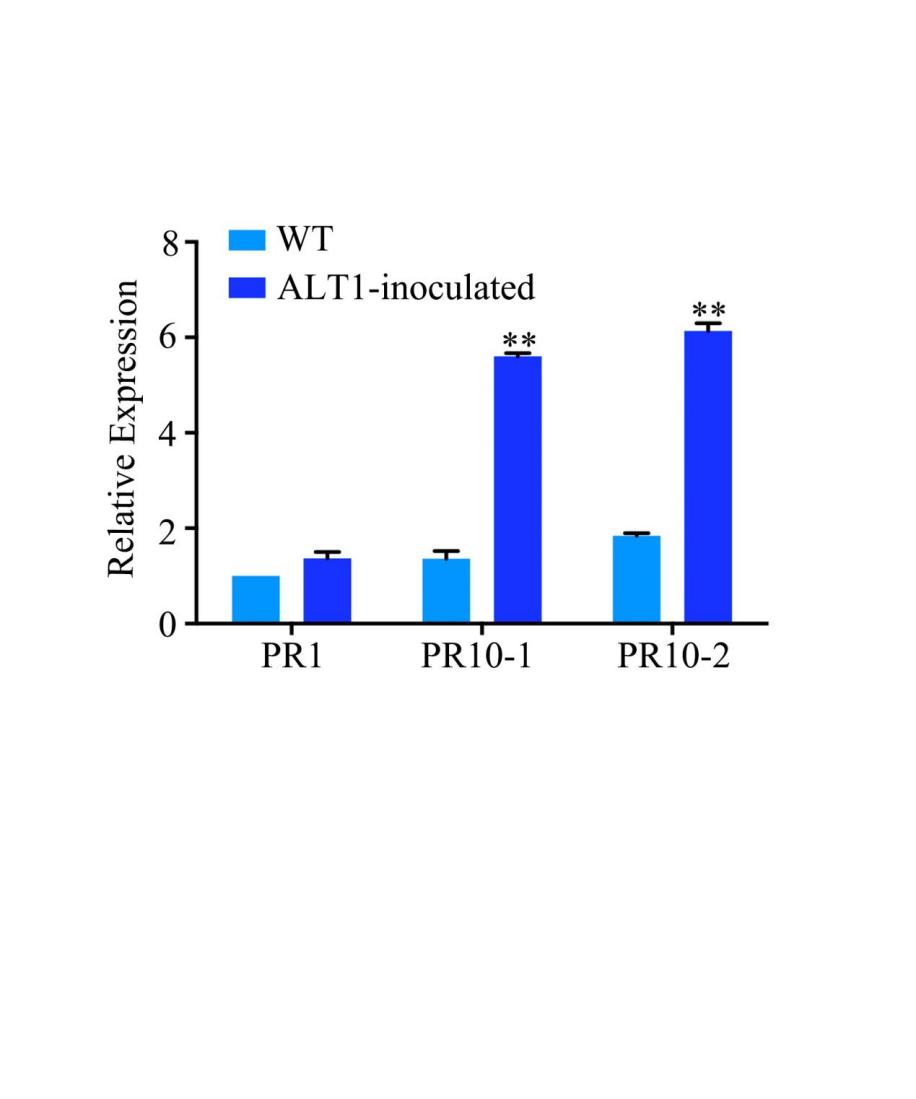


**Supplemental Figure 6. Purification of MdPR10-1 and MdPR10-2 recombinant proteins.** Lane 1: Protein marker. Lane 2: MdPR10-1 recombinant proteins purified with GST resin. Lane 3: MdPR10-2 recombinant proteins purified with GST resin.


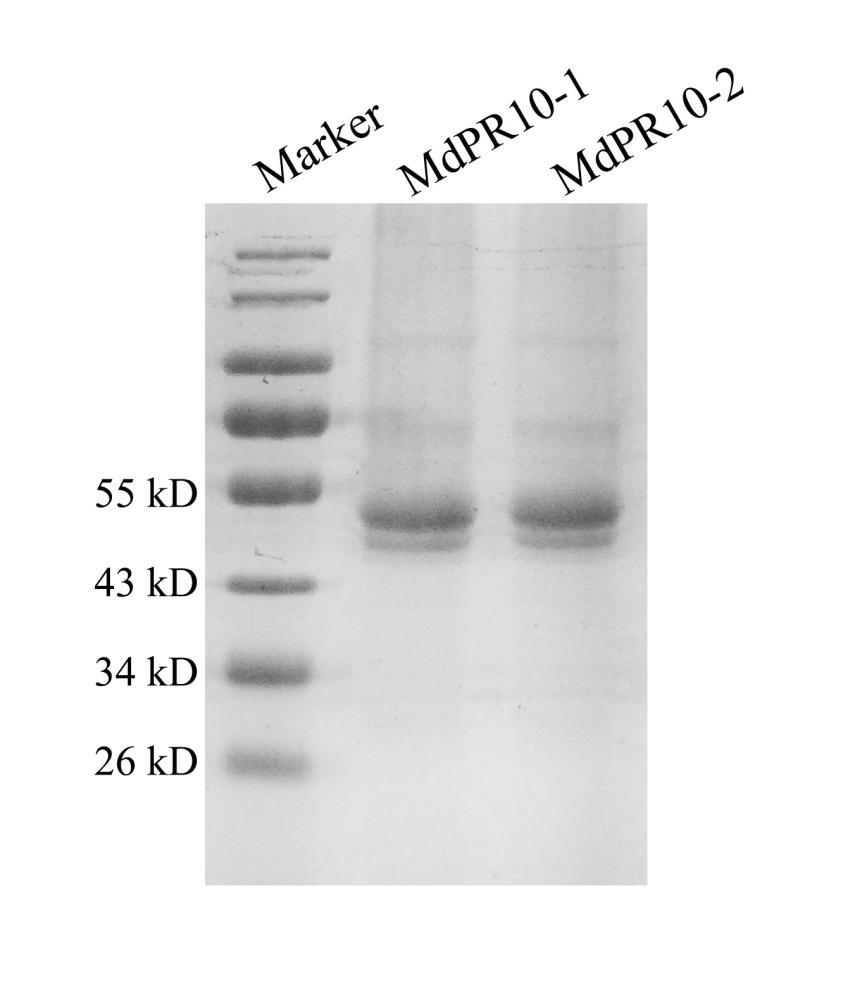


**Supplemental Figure 7. *MdPR10-1* and *MdPR10-2* expression in OE-MdPR10-1 and OE-MdPR10-2 NGR196 leaves.** *MdPR10-1* and *MdPR10-2* transcript levels in NGR196 leaves, as revealed by RT-qPCR. Error bars = SD. RT-qPCR data were calculated based on three biological and three technical replicates.


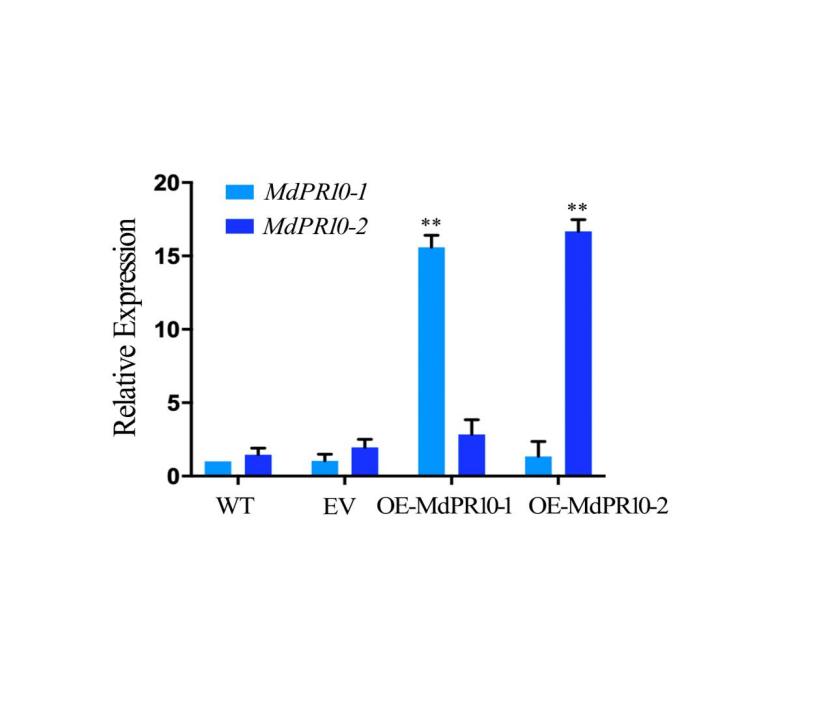


**Supplemental Figure 8. Model of a novel regulatory mechanism in which MdRNL2 and MdRNL6 interact with MdPR10-1 and MdPR10-2, inhibiting fungal growth and contributing to *Alternaria* leaf spot resistance in apple.**


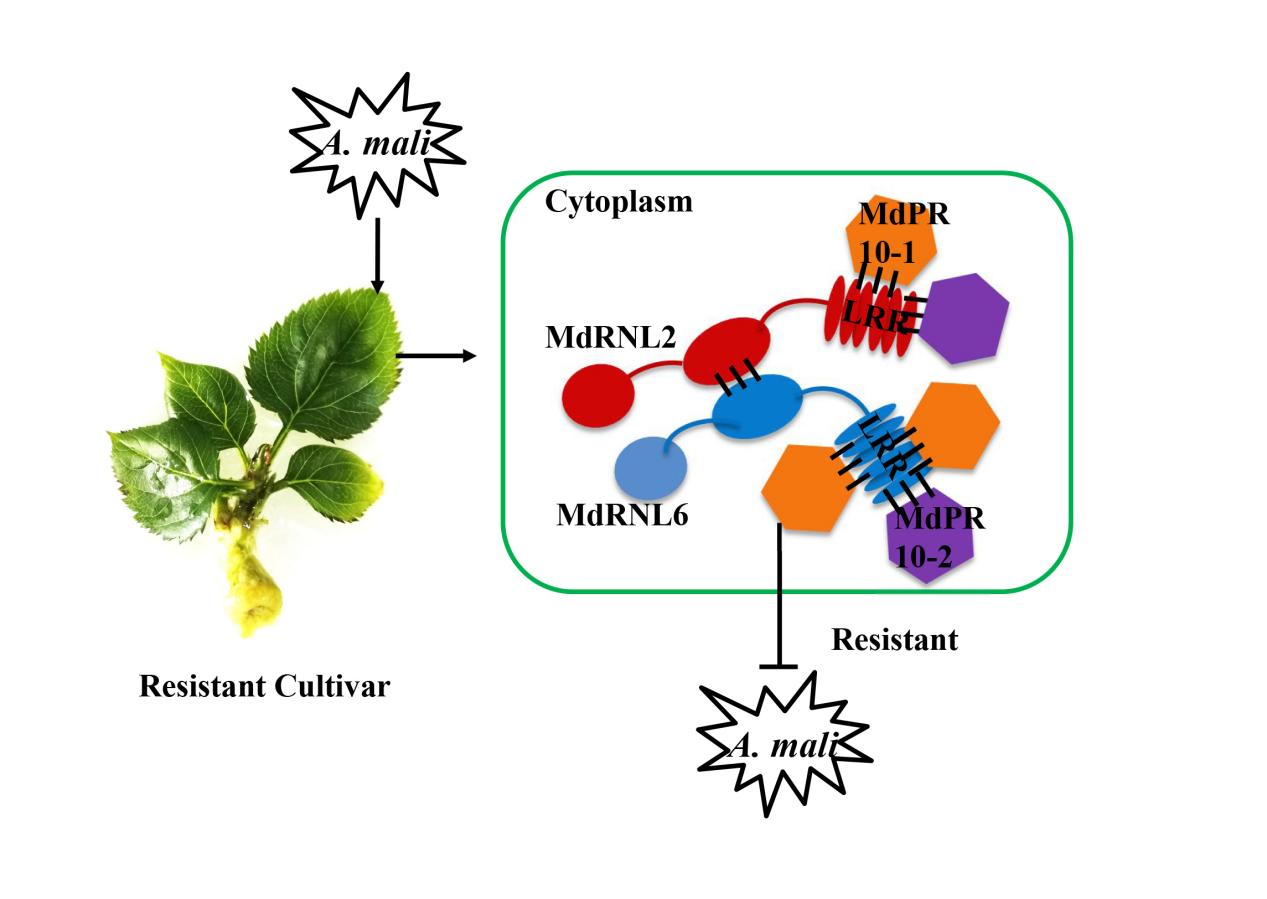


**Supplemental Table 1. Primer sequences used to clone the genes.**

| Gene name | Forward primer | Reverse primer |
| --- | --- | --- |
| MdPR10-1 | ATGGGTGTCTTCACATACGAATCCG | TTAGTTGTAGGCGTCCTGGTGCTCC |
| MdPR10-2 | ATGGGTGTCTACACATTTGAGAACG | TTAGTTGTATGCGTCGGGGTGGC |
| MdActin | TGACCGAATGAGCAAGGAAATTACT | TACTCAGCTTTGGCAATCCACATC |
| pGADT7 | CAAAGTGGGAATATTGCTGATAGC | GGGTATCTTCATCATCGAATAG |
| pGBKT7 | CAAGCATGCGATATTTGCCGAC | ATCAGTCTCCACTGAAGCCAATC |
| 1300-YFPc | CAAGATCCGCCACAACATCGAG | AACTCCAGCAGGACCATGTGATC |
| 1300-YFPn | GTAAACGGCCACAAGTTCAGCG | GAAGTCGTGCTGCTTCATGTGG |
| MdPR10-1-AD | GGATCCATATGGGTGTCTTCACATAC | CTCGAGTTAGTTGTAGGCGTCCTGG |
| MdPR10-1-BK | CCATGGCCATGGGTGTCTTCACATAC | GGATCCTTAGTTGTAGGCGTCCTGG |
| MdPR10-2-AD | GGATCCATATGGGTGTCTACACATTTG | CTCGAGTTAGTTGTATGCGTCG |
| MdPR10-2-BK | CCATGGCCATGGGTGTCTACACATTTG | GGATCCTTAGTTGTATGCGTCG |
| MdPR10-1-YFPc | GGATCCATGGGTGTCTTCACATACG | GTCGACGTTGTAGGCGTCCTGGTGC |
| MdPR10-1-YFPn | GGATCCATGGGTGTCTTCACATACG | GTCGACGTTGTAGGCGTCCTGGTGC |
| MdPR10-2-YFPc | GGATCCATGGGTGTCTACACATTTG | GTCGACGTTGTATGCGTCGGGGTGG |
| MdPR10-2-YFPn | GGATCCATGGGTGTCTACACATTTG | GTCGACGTTGTATGCGTCGGGGTGG |
| RNAi1-MdPR10-1 | CGCCATGGGGTGAAGGTAGCACATACAGC | CGATTTAAATGACGGAACCGCTGCCGGAAGCC |
| RNAi2-MdPR10-1 | CGTCTAGAGGTGAAGGTAGCACATACAGC | CGGGATCCGACGGAACCGCTGCCGGAAGCC |
| RNAi1-MdPR10-2 | CGCCATGGCAATACGGCTACGTGAAGCAC | CGATTTAAATGATGGAACCACTTCCAGATGCC |
| RNAi2-MdPR10-2 | CGTCTAGACAATACGGCTACGTGAAGCAC | CGGGATCCGATGGAACCACTTCCAGATGCC |

**Supplemental Table 2. Primer sequences used to perform RT-qPCR.**

| Gene name | Forward primer | Reverse primer |
| --- | --- | --- |
| *MdRNL2* | GCGGACGGCTATTACACTGA | GCTGAGAAGGCTCCATGTGT |
| *MdRNL6* | GAGCTCACCACCCGAAGTTT | ATTGGGTCTTGACTCGCCTG |
| *MdPR1* | GGCTCAGTCCTTATCCAATCCTC | GCCTGCTACTTTGTCATCCCACG |
| *MdPR10-1* | GCGGCGTTGGAACCATTAAG | GAGGCCTTTTCTTTGCCAGC |
| *MdPR10-2* | GATCGACTCGGTTGACGAGG | TAGTTGTATGCGTCGGGGTG |
| *MdActin* | TGACCGAATGAGCAAGGAAATTACT | TACTCAGCTTTGGCAATCCACATC |
| *AMT1* | GAAATACGCTGCAAGCCGAG | CTGGTCCCACAATTGCAACG |
