# Supplementary figures and images for: Two pathogenesis-related proteins interact with leucine-rich repeat proteins to promote Alternaria leaf spot resistance in apple
Source: Hortic Res. 2021 Oct 1;8:219. doi: 10.1038/s41438-021-00654-4 (PMC8484663; doi:10.1038/s41438-021-00654-4)

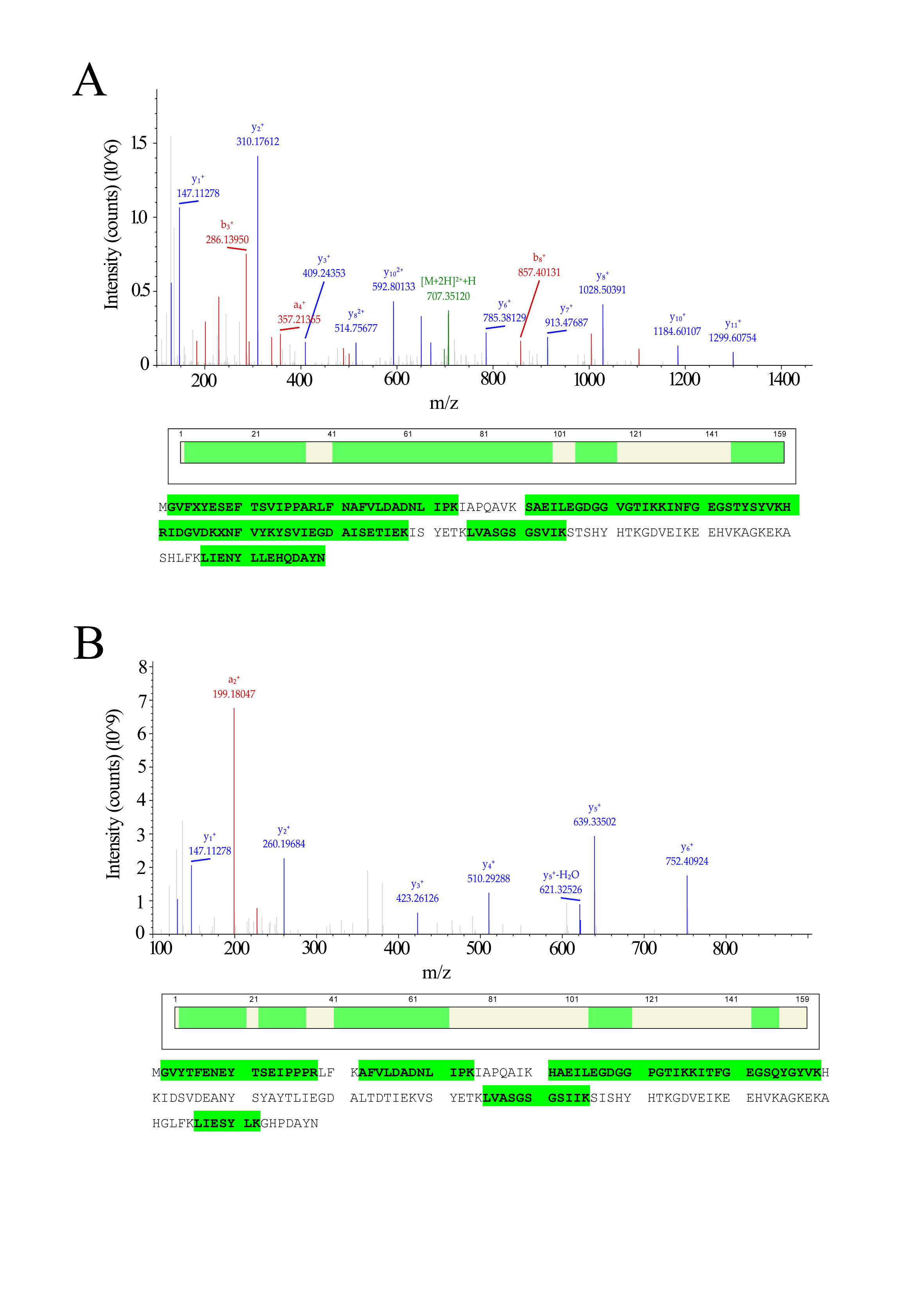

Supplement: Supplementary file 2 — Supplemental Figure 1 [file 41438_2021_654_MOESM2_ESM.jpg]

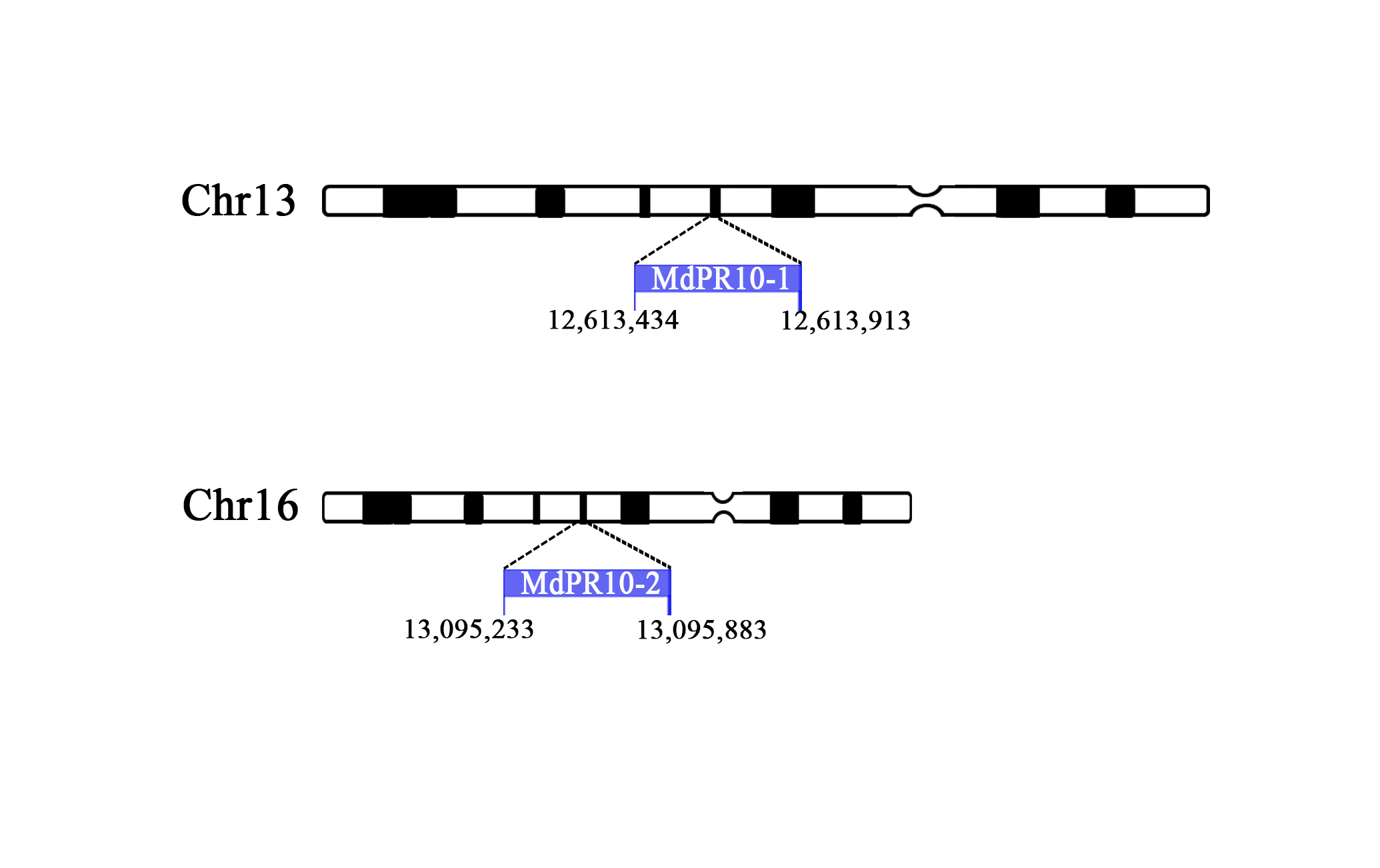

Supplement: Supplementary file 3 — Supplemental Figure 2 [file 41438_2021_654_MOESM3_ESM.jpg]

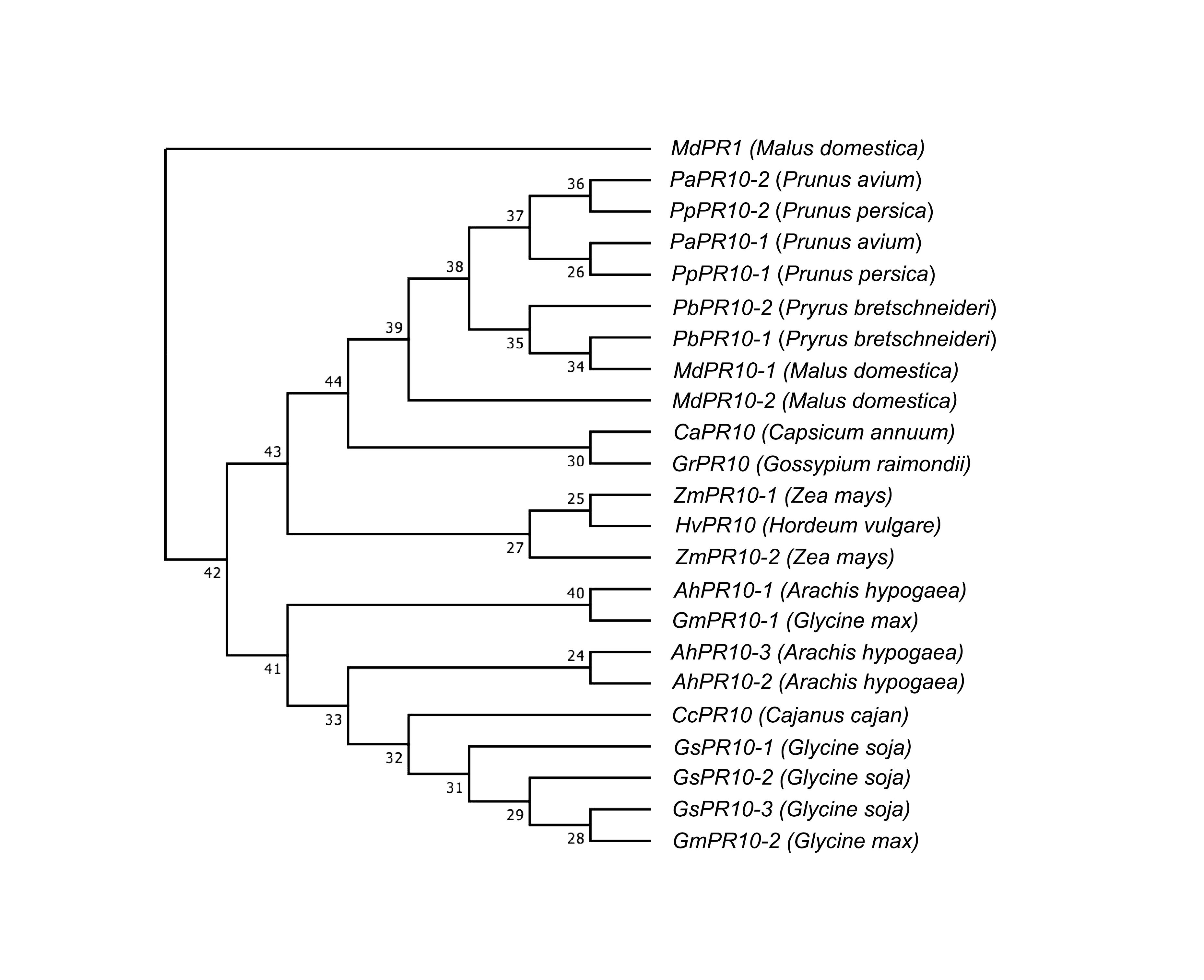

Supplement: Supplementary file 4 — Supplemental Figure 3 [file 41438_2021_654_MOESM4_ESM.jpg]

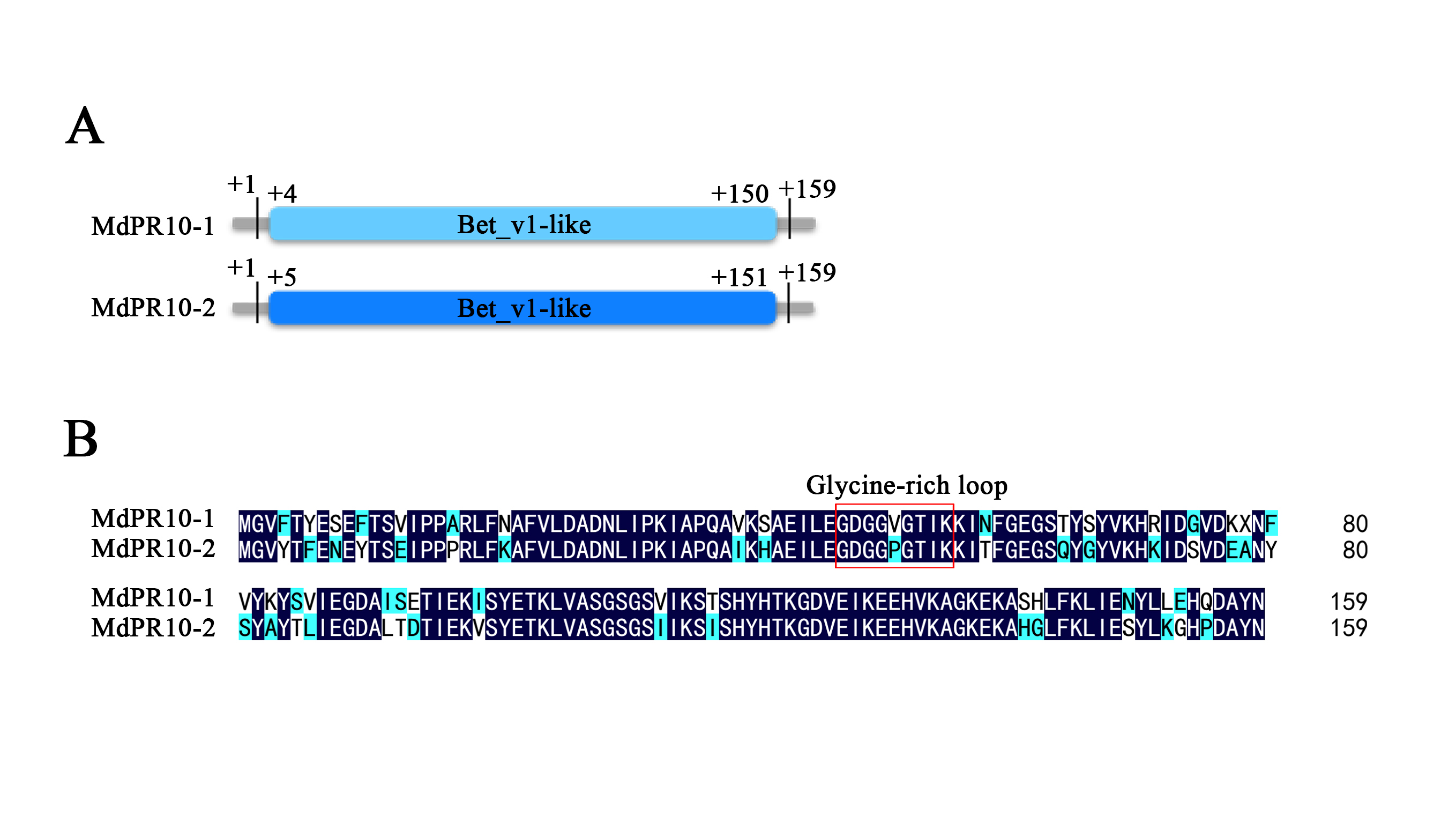

Supplement: Supplementary file 5 — Supplemental Figure 4 [file 41438_2021_654_MOESM5_ESM.jpg]

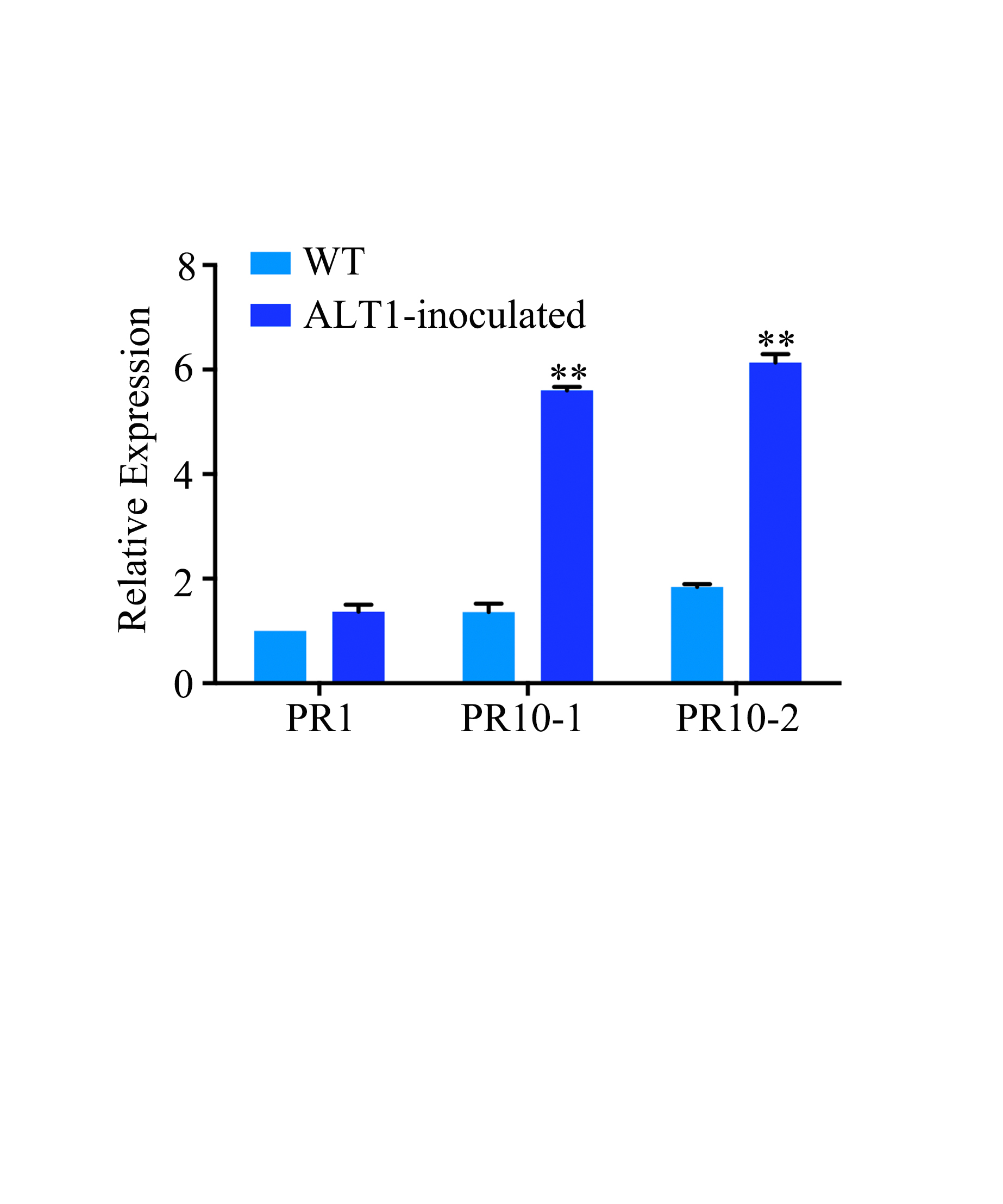

Supplement: Supplementary file 6 — Supplemental Figure 5 [file 41438_2021_654_MOESM6_ESM.jpg]

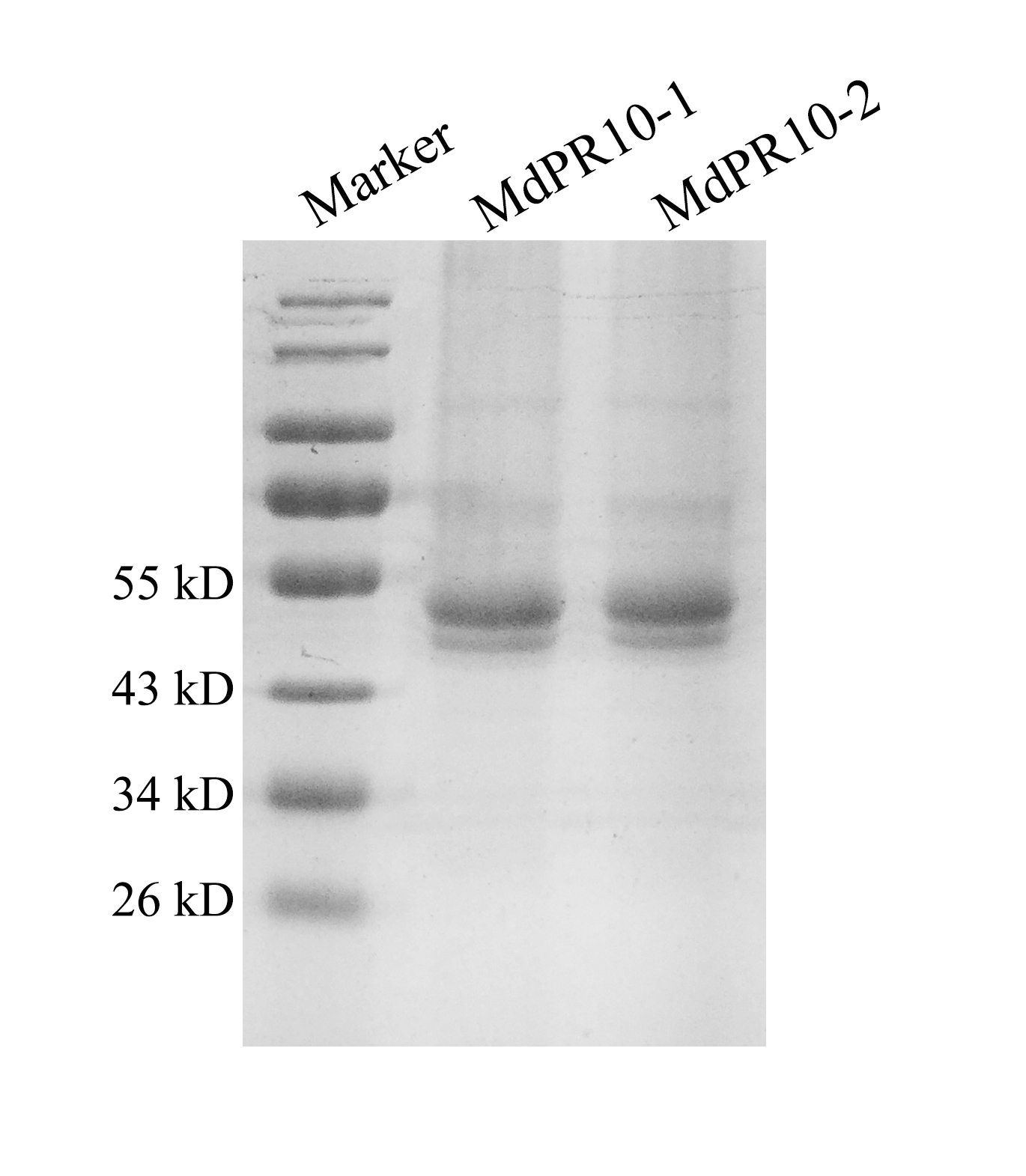

Supplement: Supplementary file 7 — Supplemental Figure 6 [file 41438_2021_654_MOESM7_ESM.jpg]

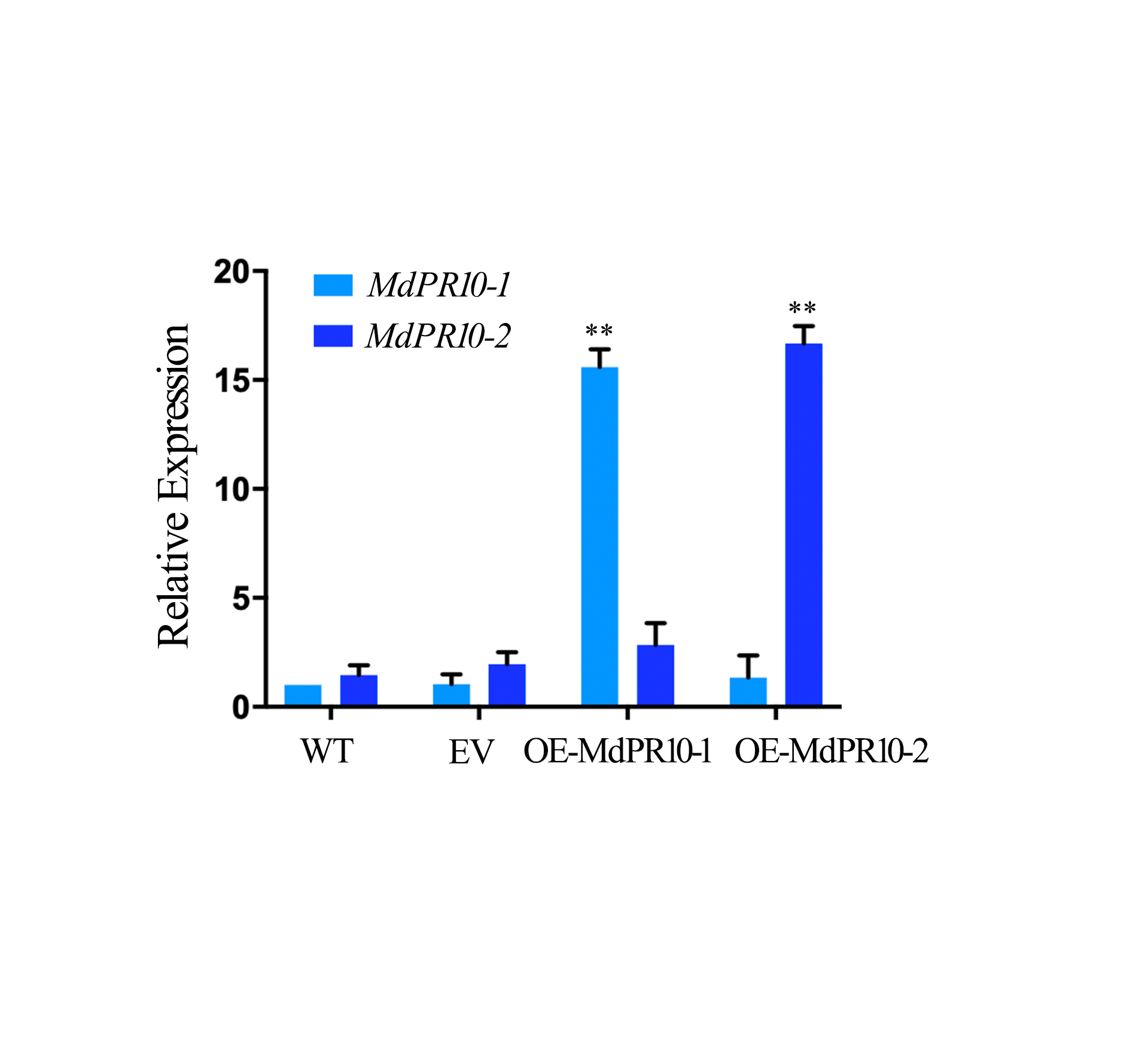

Supplement: Supplementary file 8 — Supplemental Figure 7 [file 41438_2021_654_MOESM8_ESM.jpg]

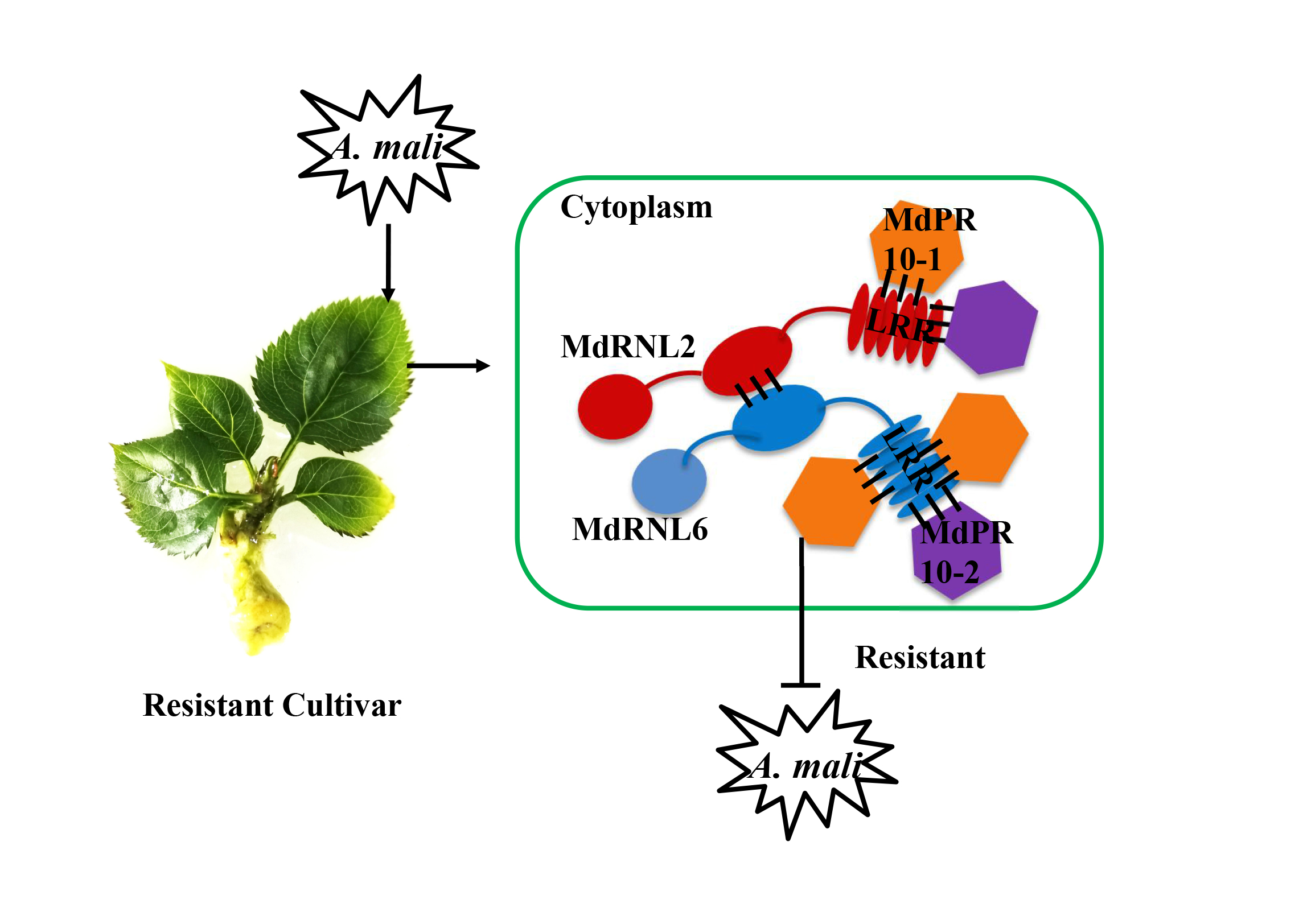

Supplement: Supplementary file 9 — Supplemental Figure 8 [file 41438_2021_654_MOESM9_ESM.jpg]
